# A new protein isoform encoded by human circular RNA circSLC8a1 contributes to cardiac remodelling

**DOI:** 10.1093/cvr/cvaf058

**Published:** 2025-04-24

**Authors:** Feiya Li, William Weidong Du, Xiangmin Li, Shuoyang Wen, Jindong Xu, Qiwei Yang, Chao Zhang, Ting Ye, Jinfeng Wei, Sheng Wang, Nan Wu, Javeria Qadir, Burton Baihua Yang

**Affiliations:** Sunnybrook Research Institute, 2075 Bayview Avenue, Toronto, ON, Canada M4N 3M5; Department of Laboratory Medicine and Pathobiology, University of Toronto, Toronto, ON, Canada M5S 1A8; Sunnybrook Research Institute, 2075 Bayview Avenue, Toronto, ON, Canada M4N 3M5; Department of Laboratory Medicine and Pathobiology, University of Toronto, Toronto, ON, Canada M5S 1A8; Sunnybrook Research Institute, 2075 Bayview Avenue, Toronto, ON, Canada M4N 3M5; Department of Laboratory Medicine and Pathobiology, University of Toronto, Toronto, ON, Canada M5S 1A8; State Key Laboratory of Applied Microbiology Southern China, Guangdong Provincial Key Laboratory of Microbial Safety and Health, National Health Commission Science and Technology Innovation Platform for Nutrition and Safety of Microbial Food, Institute of Microbiology, Guangdong Academy of Sciences, Guangzhou 510070, China; Sunnybrook Research Institute, 2075 Bayview Avenue, Toronto, ON, Canada M4N 3M5; Department of Laboratory Medicine and Pathobiology, University of Toronto, Toronto, ON, Canada M5S 1A8; Sunnybrook Research Institute, 2075 Bayview Avenue, Toronto, ON, Canada M4N 3M5; Department of Laboratory Medicine and Pathobiology, University of Toronto, Toronto, ON, Canada M5S 1A8; Department of Anesthesiology, Guangdong Cardiovascular Institute, Guangdong Provincial People’s Hospital & Guangdong Academy of Medical Sciences, Guangzhou, Guangdong Province, China; Sunnybrook Research Institute, 2075 Bayview Avenue, Toronto, ON, Canada M4N 3M5; Department of Laboratory Medicine and Pathobiology, University of Toronto, Toronto, ON, Canada M5S 1A8; Sunnybrook Research Institute, 2075 Bayview Avenue, Toronto, ON, Canada M4N 3M5; Department of Laboratory Medicine and Pathobiology, University of Toronto, Toronto, ON, Canada M5S 1A8; Sunnybrook Research Institute, 2075 Bayview Avenue, Toronto, ON, Canada M4N 3M5; Department of Laboratory Medicine and Pathobiology, University of Toronto, Toronto, ON, Canada M5S 1A8; Department of Anesthesiology, Guangdong Cardiovascular Institute, Guangdong Provincial People’s Hospital & Guangdong Academy of Medical Sciences, Guangzhou, Guangdong Province, China; Department of Anesthesiology, Beijing Anzhen Hospital, Capital Medical University, Beijing, China; Sunnybrook Research Institute, 2075 Bayview Avenue, Toronto, ON, Canada M4N 3M5; Department of Laboratory Medicine and Pathobiology, University of Toronto, Toronto, ON, Canada M5S 1A8; Sunnybrook Research Institute, 2075 Bayview Avenue, Toronto, ON, Canada M4N 3M5; Department of Laboratory Medicine and Pathobiology, University of Toronto, Toronto, ON, Canada M5S 1A8; Sunnybrook Research Institute, 2075 Bayview Avenue, Toronto, ON, Canada M4N 3M5; Department of Laboratory Medicine and Pathobiology, University of Toronto, Toronto, ON, Canada M5S 1A8; Institute of Medical Sciences, University of Toronto, Toronto, ON, Canada M5S 3H2

**Keywords:** CircRNA, CircSLC8a1, CircRNA translation, Heart function, Mitochondria, ATP synthesis

## Abstract

**Aims:**

Circular RNA circSLC8a1 has been previously suggested to possess translation potential, but experimental evidence supporting this notion has been lacking. We aim to understand the functions of circSLC8a1 and its translated protein in cardiac remodelling.

**Methods and results:**

To elucidate the functional significance of circSLC8a1, we established a transgenic mouse line expressing circSLC8a1 and its translated protein SLC8a1-604. We present compelling evidence confirming the translation potential of circSLC8a1 (hsa_circ_0005232) both *in vitro* and *in vivo*. The back-splicing event within hsa_circ_0005232 leads to the generation of a novel circRNA-derived protein comprising 604 amino acids, named SLC8a1-604, which has not been previously reported. These SLC8a1-604 transgenic mice exhibited a heart failure phenotype. In further investigations, we induced pressure overload in the transgenic mice, revealing a significant decrease in heart function compared to litter-matched negative controls. Notably, our findings indicate that the reduced heart function observed in the transgenic mice can be attributed to the presence of the circRNA-translated protein, SLC8a1-604, rather than the circRNA itself. Mechanistically, we found that SLC8a1-604 translocated into mitochondria, where it exerted its effects by binding to POLRMT. This interaction results in a downregulation of mitochondrial gene transcription, leading to a decrease in ATP synthesis.

**Conclusion:**

Our study provides evidence that circSLC8a1 has the capacity to encode a novel protein isoform, SLC8a1-604, which plays a pivotal role in the regulation of heart functions: circSLC8a1 modulates the remodelling process of cardiac pressure overload by translating into a functional protein.

## Graphical Abstract

**Graphical Abstract:**
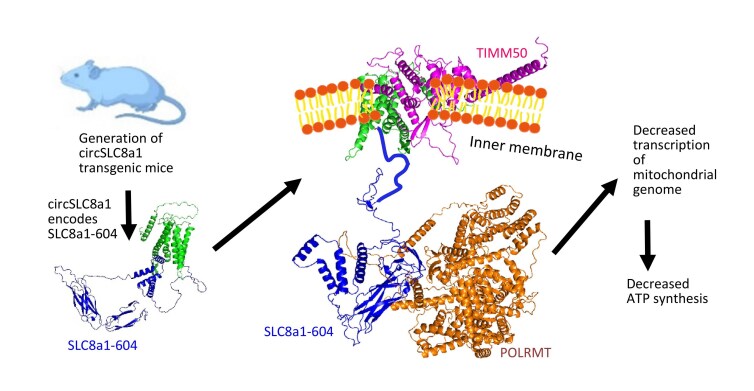

**Time of primary review: 41 days**

## 1. Introduction

Translational regulation plays a pivotal role in gene expression, yet our comprehension of its functions within human heart tissues remains incomplete. Ribosome profiling, also known as Ribo-seq, offers a powerful approach to characterize mRNA footprints, enabling us to predict ribosome movement based on these footprints. This information is further employed to investigate open reading frames (ORFs). With this technology, ORFs have been extensively detected in non-coding RNAs producing functional peptides. When they were initially detected, circRNAs were considered as non-coding RNAs. Similar to the other non-coding RNAs, circRNAs were reported to function as microRNA sponge and/or by interacting with proteins.^1–6^ Starting in 2017, some circRNAs have been reported to be translated, producing new protein isoforms.^7–9^ Due to the smaller sizes of circRNAs relative to their cognate linear mRNAs, circRNAs usually produce smaller proteins, since they are most likely generated from partial ORF of the cognate linear mRNAs. To date, circRNA translation has shown several features, different from the cognate linear mRNA translation.^10,11^ For example, (i) circNlgn is generated by the second exon of the *Nlgn* gene, and the translation of circNlgn extends beyond the back-splicing junction (BSJ) for an additional nine amino acids, resulting in a peptide that acquires novel function in the regulation of cardiac remodelling, fibrosis, and colitis progression.^12–14^ (ii) If the translation of the circRNAs generates partial sequences of cognate full-length proteins, and a stop codon is created by back-splicing, the translated proteins are still regarded as novel protein isoforms, since such a polypeptide cannot be generated by the cognate full-length mRNAs.^15^ Many circRNA translation has this feature.^16–20^ (iii) Circularization of exons 14 and 15 of epidermal growth factor receptor (EGFR) mRNA generated circEGFR that shows infinite open reading frame resulting in the production of repeating amino acid sequences via rolling translation.^21^ (iv) In addition, a specific N-terminal extension can be obtained due to the ORF frameshifting. The start codon of circFNDC3B-218aa is located at nt-404 downstream of the BSJ that generates a novel protein fragment (41 amino acids). Translation continues until it encounters a stop codon and produces an N-terminal extension.^22^ CircRNA can also regulate the translation of parental genes.^23^

The translation of circSLC8a1 appears to differ between humans and mice. A paper published in *Cell*, conducted by van Heesch and colleagues in 2019, identified 40 circular RNAs with potential protein-coding activity based on an analysis of 80 human heart translatomes.^24^ In the study, ribosome profiling revealed several ribosome-associated circRNAs, including circSLC8a1, suggesting its protein-coding potential in human circSLC8a1. In contrast, another study published in *Cardiovascular Research*, performed by Lim and colleagues in 2019, indicated that mouse circSLC8a1 is unlikely to be translated but instead exerts functional roles through a miRNA sponging mechanism.^25^ Our study generating mouse circSLC8a1-transgenic mice detected a potent cardiac phenotype,^26^ but no protein translation was observed (data not shown). In both human and mouse circRNAs, circSLC8a1 originates from the large second exon of solute carrier family 8 member 1 (NCX1). The sodium–calcium exchanger gene SLC8a1 plays a critical role in mediating the exchange of one calcium ion (Ca2+) and three to four sodium ions (Na+) across the cell membrane. This function is essential for maintaining calcium homeostasis in the cytoplasm and thereby regulating Ca2+-dependent cellular processes. When there is a rapid change in cytoplasmic Ca2+ levels, often initiated by the opening of voltage-gated channels and the release of Ca2+ from intracellular stores in the endoplasmic reticulum (ER), SLC8a1 regulates the return of Ca2+ levels to a steady state by facilitating the removal of Ca2+ from the cell. Since circSLC8a1 is one of the highly expressed circular RNA in human heart tissues, studies have been conducted trying to illustrate its functions in physiological and pathological conditions. These studies provided early evidence showing that the expression of circSLC8a1 plays important roles in regulating heart functions.^25,27^ However, the regulatory roles of circSLC8a1 and its clinical relevance have been debated between these studies. There is currently no direct evidence showing that the functions of circSLC8a1 are mediated by its translated protein: it is even not clear whether or not circSLC8a1 can be translated. Here, we provided the first evidence showing the existence of a new isoform SLC8a1-604 translated from circSLC8a1. To understand the functions of circSLC8a1 and its translated protein, we generated a transgenic mouse line that stably expressed circSLC8a1 to study the physiological and pathological roles of circSLC8a1. Our study demonstrates the important roles of circSLC8a1 in the cardiac pressure-overload remodelling process by translating it into a functional protein. This proof-of-concept study may lay the foundation for potential clinical applications in circular RNA therapy.

## 2. methods

### 2.1 Construct generation and primer design

The expression constructs of circSLC8a1 were generated according to the sequences shown in the Supplementary Information (DNA/RNA Sequences). The plasmid vector contains a Bluescript backbone, with one cytomegalovirus (CMV) promoter driving the green fluorescent protein (GFP) expression, and another CMV promoter driving the circRNA-forming fragments. For the vector control, the circSLC8a1 insert sequence was replaced with a non-related random sequence. The constructs of pre-circSLC8a1 (Precursor) and mut-circSLC8a1 (circ-mut) were generated in which either the intron sequences for the circularization signal were deleted or the translation initiation signal was mutated. Thus, pre-circSLC8a1 would express the linear transcripts without circularization, and mut-circSLC8a1 would express the circRNA without being translated to a peptide. The sequences of all the constructs, primers, and siRNAs are listed in the Supplementary Information (see Supplementary material online, Figures  *S1*-*5*).

### 2.2 Animal models

Animal experiments were carried out in accordance with the guidelines and regulations approved by the Animal Care Committee of Sunnybrook Research Institute. All procedures conformed to the guidelines from the NIH Guide for the Care and Use of Laboratory Animals. The circSLC8a1-transgenic mouse line was generated through the pronuclear microinjection of DNA fragments containing circSLC8a1 into C57BL/6J. The microinjection was performed by The Centre for Phenogenomics, Toronto. Each transgenic mouse was ear-tagged and genotyped. The genotyping primer sequences are listed in the Supplementary Information.

Isoflurane was administrated as an inhalant anaesthetic during the mouse surgery. Anaesthesia was induced in an isoflurane induction chamber and maintained using a nose cone delivering a mixture of isoflurane and oxygen. Isoflurane (1.5–2%) was delivered at a flow rate of 0.4–0.8 litres/min.

We induced a pressure-overload (PO) heart failure model using a modified transverse aortic constriction (TAC) procedure in mice, as previously described.^12^ Successful banding was confirmed by measuring carotid artery flow velocities via Doppler ultrasound. Only mice with a right carotid (RC)/left carotid (LC) flow ratio exceeding 4 were included in subsequent experiments. Sham mice underwent anaesthesia and underwent all surgical steps except aortic banding.

For circSLC8a1 injected TAC model, 8-week-old C57BL6 mice underwent TAC and were randomly grouped (mixed gender) and injected intraperitoneally with circSLC8a1 and other plasmids (50 μg/each) twice a week for 6 weeks. Ten mice were used in each experimental group, except as mentioned in the figure legends. The plasmids were conjugated with mPEG and gold nanoparticles (AuNP) that formed complexes before injection. A group of sham and TAC mice without injection were used as controls. Six weeks after injection, all mice were euthanized by cervical dislocation following cardiac function assessment. The hearts were harvested and bisected. The upper half of the heart was kept frozen for PCR or processed to obtain frozen sections, followed by immunofluorescence and circSLC8a1 hybridization staining. The lower half was fixed with 10% buffered formalin and embedded in paraffin, then sectioned for Masson’s Trichrome staining and Sirius red staining.

### 2.3 RNA sequencing

Total RNAs were extracted from both circSLC8a1-transgenic and litter-matched negative counterpart mice. The standard procedure for mRNA enrichment and fragmentation was followed. Reverse transcription was then carried out to synthesize cDNA, which was subsequently PCR-amplified and used to generate libraries for sequencing. These sequencing procedures were conducted by Novogene (www.novogene.com) using the NEBNext® Ultra™ Directional RNA Library Prep Kit for Illumina® (NEB, USA, Catalog #: E7420).

### 2.4 Synthesis and delivery of circSLC8a1 plasmid-polyethylene glycol (PEG)-Au NP

To synthesize the circSLC8a1 plasmid and siRNA–PEG conjugates, 20 nmol of plasmids or thiol-modified siRNAs were dissolved in 800 µL of RNase-free water. mPEG-SH (PG1-TH-2k, Nanocs, New York, NY) was combined with the plasmid at a 1:20 molar ratio. Additionally, 10 nm gold nanoparticles (Cytodiagnostics, Burlington, Ontario, Canada) were mixed with the plasmid or siRNA–PEG conjugate at a weight ratio of 1:1 for conjugation. The resulting mixture was gently shaken at 60°C for 30 min and then transferred into a syringe. Subsequently, the circSLC8a1 plasmid-PEG-Au NP or siRNA–PEG-Au NP conjugates were administered intraperitoneally in a volume of 100 µL, as previously described.^26^

### 2.5 Isolation of primary mouse cardiomyocytes (PCMs) and primary cardiac fibroblasts (PCFs)

To isolate primary mouse cardiomyocytes (PCMs) and primary cardiac fibroblasts (PCFs) from neonatal mice, the animals were sacrificed by cervical dislocation. The hearts were rapidly removed and washed in PBS with 20 mM 2,3-butanedione monoxime (BDM), transferred into a drop of 4-(2-Hydroxyethyl)piperazine-1-ethanesulfonic acid (HEPES)-buffered Tyrode solution containing (mM): 130 NaCl, 5.4 KCl, 1 CaCl_2_, 1 MgCl_2_, 0.33 Na_2_HPO_4_, 10 HEPES, 5.5 glucose (pH of 7.4), and minced into small pieces. Cardiac tissues were fragmented and incubated in 25 mL Tyrode solution (0.012 g Collagenase D, 0.009 g Collagenase B, and 0.001 g Protease XIV from Streptomyces griseus) at 37°C for 20–30 min. Digested products were filtered and centrifuged at 600 rpm for 5 min. The cell pellet was resuspended in Dulbecco's Modified Eagle Medium (DMEM)/F12 medium with 10% fetal bovine serum (FBS) and 20 mM BDM, plated onto a 10-cm cell culture dish, and incubated for 1–3 h in a cell culture incubator. This pre-plating step isolated PCFs, which adhered to the uncoated cell culture dish. Non-adherent cells were transferred to a cell culture dish coated with 1% gelatin solution to harvest PCMs. The isolated PCFs were cultured in 10% FBS/DMEM, and PCMs were maintained in DMEM/F12 medium with 10% FBS and 1 mM glutamine.

### 2.6 Pull down assays

SLC8a1 (NCX1) antibody was used to immune-precipitate 293T cells transfected with the vector or circSLC8a1 plasmids. The transfected cells were collected and washed twice with PBS and lysed with CO-IP buffer (with 1% proteinase inhibitor cocktail). Samples were further sonicated, and the concentrations were balanced. 5% of each sample was saved as input. The other samples were subjected to incubation with magnetic beads pre-incubated with SLC8a1 antibody at room temperature for 1 h. The beads were further incubated at room temperature for 1 h, followed by three times washing with the CO-IP buffer to get rid of non-specific binding. The washed beads were eluted with 1× loading dye and incubated at 95°C for 10 min. The eluted proteins were subjected to further analysis.

The mammalian expression construct circSLC8a1-His was transiently transfected into 293T cells. Proteins prepared from the cell lysate were subjected to His-tagged protein pull-down with Ni-NTA Agarose and eluted according to the manufacturer’s instructions. The eluted proteins were subjected to LC-MS/MS or Western blotting.

### 2.7 Sucrose gradient assay

Sucrose gradient assay was performed to determine the translation of circSLC8a1. Polysomes were extracted from the cells transfected with the vector or circSLC8a1 plasmid with or without 100 µg/mL puromycin treatment for 10 min prior to harvest. Polysomes were prepared in hypotonic buffer containing 5 mM Tris–HCl (pH, 7.5), 2.5 mM MgCl_2_, 1.5 mM KCl, 1x protease inhibitor cocktail (EDTA-free), 0.5% Triton X-100, 0.1 mg/mL cycloheximide, and 0.5% sodium deoxycholate. Sucrose was filled in ultracentrifuge tubes to obtain a 10–50% gradient with sucrose solutions. The polysomes-containing mix was collected after brief centrifugation for 10 min and loaded on top of the sucrose gradient solution followed by ultracentrifugation at 30,000 rpm at 4°C for 2 h. The polysome fractions were collected from top to bottom. The absorbance at 254 nm (A254 nm) was measured and RNA extraction from each fraction was performed. The RNA levels of circLSC8a1 and GAPDH (internal control) in each fraction were determined by real-time PCR.

### 2.8 Mass spectrometry analysis

HEK293T cells transfected with the vector or circSLC8a1 were lysed in Co-IP buffer and applied for either circRNA pull-down assay with the circSLC8a1 probe following the methods described above or protein pull-down assay with the SLC8a1 antibody. The magnetic beads were collected and washed extensively with the Co-IP buffer followed by washing with PBS. The magnetic beads were sent to the SPARC Molecular Analysis Centre (The Hospital for Sick Children, Toronto) for elution, digestion, and mass spectrometry analysis.

### 2.9 Human heart specimens

The study was carried out in accordance with The Ethics Code of the World Medical Association (Declaration of Helsinki). The use of human samples was approved by the Ethics Committee of Guangdong Provincial People’s Hospital. Patients in this study gave formal informed consent prior to enrolment. Heart samples were obtained from heart donations or surgeries from 20 patients with heart failure and 20 people without heart disease records.

### 2.10 Bioinformatics of protein docking and mitochondrial targeting

3D Structure of TIMM50 (Q3ZCQ8) and POLRMT (O00411) were downloaded from AlphaFold Protein Structure Database (https://alphafold.ebi.ac.uk). 3D Structure of SLC8a1-604 was predicted using the online server Robetta (https://robetta.bakerlab.org/submit.php).^28^ Interaction of SLC8a1-604 with TIMM50 and POLRMT was predicted using the online server LZerD Doking (https://lzerd.kiharalab.org/upload/),^29^ and the best models were viewed in PyMOL version 2.5.5.

The cleavage site of mitochondrial processing peptidase (MPP), the score region of Max positively charged amphiphilicity (PA), and the TOMM20 recognition motif of SLC8a1-604 were predicted using the online server MitoFates (https://mitf.cbrc.pj.aist.go.jp/MitoFates/cgi-bin/top.cgi).^30^

The general methods for Cardiac function assessment, Subcellular fractionation, Western blotting, ATP assay, Histological staining, Fluorescence *in situ* Hybridization (FISH), and Immunofluorescence staining were performed as previously described.^31–38^ The specific details are provided in the Supplementary Information.

### 2.11 Statistical analysis

All experiments were conducted in triplicate unless otherwise specified in the figure legends. The normality of the data distribution was assessed using the Shapiro-Wilk test. Data were presented as the mean (represented by bars) with the standard deviation (indicated by whiskers). For comparisons between two groups with a single independent factor, a two-tailed unpaired Student’s *t*-test was employed. In cases involving multiple group comparisons, a one-way ANOVA followed by a Bonferroni post hoc test was applied for the analysis of one independent variable. Prism 8 software (GraphPad Software, La Jolla, CA) was utilized for the aforementioned statistical analyses, and statistical significance was determined at *P* < 0.05.

## 3. Results

### 3.1 Human circSLC8a1 encodes a protein SLC8a1-604

**Figure 1:**
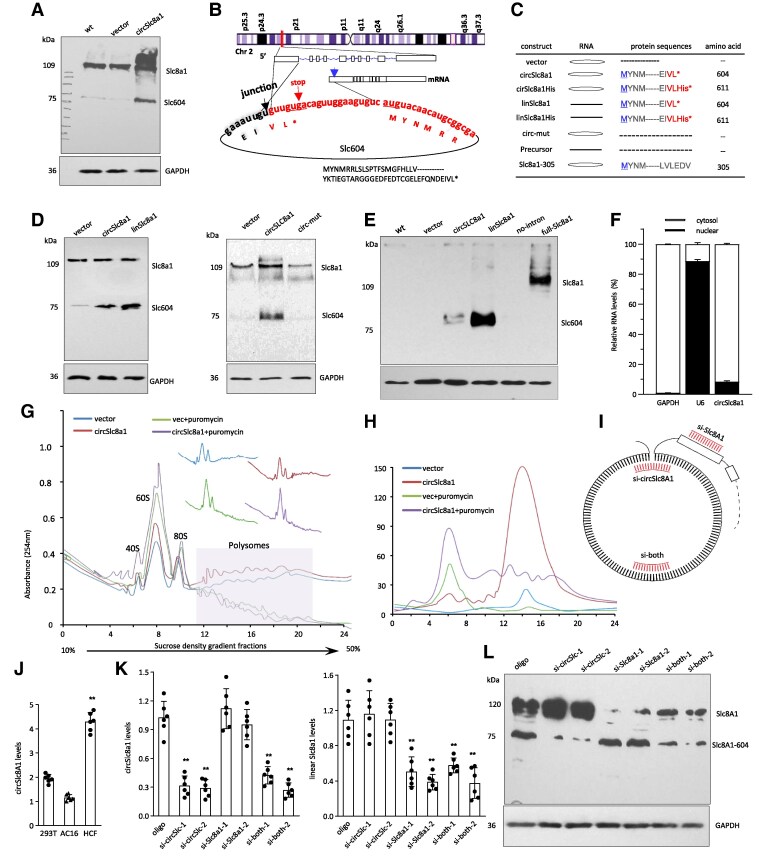
Translation of circSLC8a1. (*A*) Transfection of 293T cells with the circSLC8a1 plasmid showed an increased level of SLC8a1-604. (*B*) CircSLC8a1 is generated from Exon 2 of SLC8a1 containing 1829 nt, while the full-length SLC8a1 mRNA contains 21 029 nt. CircSLC8a1 harbours the translation initiation unit of SLC8a1 and can potentially be translated into 604 amino acids (SLC8a1-604). (*C*) Structure of the constructs used in the study. (*D*) Left, overexpression of circSLC8a1 in 293T cells showed a clear band similar to that produced by linSLC8a1 plasmid. Right, no band was detected when the translation initiation was mutated (circ-mut). (*E*) Upper, Western blot from left to right, WT, vector, circSLC8a1, linSLC8a1, Precursor, full-SLC8a1. The No-circ plasmid did not show a clear band. The full-SLC8a1 showed a band at 109 kDa. Lower, longer exposure time. (*F*) Fractionation was performed to identify the localization of circSLC8a1. GAPDH and U6 were used for quality control. While GAPDH was mainly detected in the cytosol and U6 in the nucleus, circSLC8a1 was mainly located in the cytosol, *n = 3*. (*G*) Sucrose gradient was performed using 293T cells transfected with the vector or circSLC8a1, with or without puromycin treatment. Treatment with puromycin inhibited polysome binding. (*H*) Lysates prepared from the vector- and circSLC8a1-transfected 293T cells were subjected to sucrose gradient assay. CircSLC8a1-transfected cells expressed higher levels of circSLC8a1 (red arrow) than the vector control (blue arrow), and most circSLC8a1 accumulated in the polysome fractions, which shifted to the lighter fractions following puromycin treatment. (*I*) A diagram illustrating the design of siRNAs targeting circSLC8a1 (si-circSlc8a1), linear SLC8a1 mRNA (si-SLC8a1), and both circSLC8a1 and SLC8a1 (si-both). (*J*) RT-PCR was performed on lysates from AC16, 293T, and human cardiac fibroblasts (HCF) cells. Among these cell types, HCF cells exhibited the highest expression level of circSLC8a1. *n* = 6, **P < 0.01. (*K*) HCFs were transiently transfected with the aforementioned siRNAs, and RT-PCR analysis revealed significant silencing of circSLC8a1 (left) and linear SLC8a1 mRNA (right) by the siRNAs targeting them. **P < 0.01 vs. oligo (*n = 6*). (*L*) Western blotting was conducted on transfected HCFs. The results showed that siRNA-mediated silencing of circSLC8a1 reduced SLC8a1-604 levels, silencing SLC8a1 reduced SLC8a1 protein levels, and transfection with siRNAs targeting both circSLC8a1 and linear SLC8a1 led to decreased levels of both SLC8a1-604 and SLC8a1 proteins. Data in (*J* and *K*) were analysed by a one-way ANOVA followed by a Bonferroni post hoc test.

Human and mouse solute carrier family 8 member A1 (*SLC8a1*) genes can generate circular RNAs circSLC8a1 that is highly conserved between both the species (88% homology). Both circRNAs result from back-splicing of Exon 2 of *SLC8A1* and contain the translation initiation codon of SLC8a1 mRNA. While examining the roles of mouse circSLC8a1, we tested whether or not mouse circSLC8a1 could translate a protein by ELISA, dot blot, and Western blot using different concentrations of SDS-PAGE and agarose gel probed by anti-SLC8a1 antibody targeting the N-terminus of the SLC8a1 protein (also called sodium–calcium exchanger 1, NCX1, Abcam, Ab177952). We could not obtain convincing results to show that mouse circSLC8a1 was translated. However, the cell lysate harvested from human 293T cells transfected with circSLC8a1 expression construct engineered by using actin intron-100 overexpression system described previously^12^ revealed a protein band of 75 kDa (*Figure 1A*). The structure and sequence of circSLC8a1 is provided in Supplementary material online, Figure  *S1*. Human circSLC8a1 is one nucleotide shorter than the mouse circSLC8a1 (1829 nt vs. 1830 nt). This created a stop codon immediately passing the back-splicing junction (*Figure 1B*). Since 293T is a human cell line, endogenous expression of the peptide was also detected in wild-type (WT) and vector-transfected cells.

Another plasmid was designed by expressing the circSLC8a1 ORF in pcDNA3.1 plasmid ensuring successful translation of the ORF (linSlc8a1, *Figure 1C*, structure and sequence provided Supplementary material online, Figure  *S2*). The expressed protein was analysed on Western blot, and a band of same size was detected, suggesting the translation potential of circSLC8a1 (*Figure 1D*, left). To further confirm the translation of circSLC8a1, we generated a translation mutation in circSLC8a1 producing circ-mut (see Supplementary material online, Figure  *S3*). This mutation construct was able to generate circRNA but failed to translate a peptide (*Figure 1D*, right). To test whether circularization is essential for circLSC8a1 translation, we generated a construct with a mutation in the 5’ intron (Precursor), the essential component in back-splicing (see Supplementary material online, Figure  *S3*). Transfection with circSLC8a1 showed a clear product at the designated protein size, while transfection with the Precursor plasmid did not show any obvious band (*Figure 1E*), showing that the precursor of circSLC8a1 was not translated. We also transfected 293T cells with a SLC8a1 full-length expression construct. A major band at 109 kDa, together with some weaker bands, was detected on the Western blot (*Figure 1E*). There were no smaller bands, implying the specificity of the antibody. These results confirmed that the 75-kDa protein band was obtained from the circSLC8a1 construct.

To corroborate these results, we measured the localization and levels of the endogenous circSLC8a1 by fractionating the cytosol and the nuclei. The experiment showed that more than 90% of circSLC8a1 was detected in the cytosol (*Figure 1F*). As the controls, GAPDH was mainly detected in the cytosol and U6 was mainly detected in the nuclei. Since ribosome association is essential for circRNA translation and sucrose gradient is a procedure to examine polysome profiling which is a common technique to examine the overall degree of translation, we performed sucrose gradient isolation of ribosome-associated RNAs with and without puromycin treatment. Polysome profiles of each group (vector group, circSLC8a1 overexpression group, puromycin treated vector group, and puromycin treated circSLC8a1 overexpressing group) were performed (*Figure 1G*). Following quantitative PCR, a significantly higher peak in the circSLC8a1-transfected cells compared to the vector control was detected within the polysome binding recognition area and both the peaks shifted to lighter polysomes after puromycin treatment, indicating inhibited translation following puromycin treatment (*Figure 1H*).

To validate the translation of circSLC8a1, we designed three sets of siRNAs to specifically target circSLC8a1, SLC8a1, or both (*Figure 1I*). Each set contains two siRNAs (see Supplementary material online, Figure  *S5B*). We examined the endogenous levels of circSLC8a1 and found that primary human cardiac fibroblasts (HCFs, sourced from ScienCell Research Laboratories, Carlsbad, CA, USA) exhibited the highest circSLC8a1 expression compared to AC16 and 239T cells (*Figure 1J*). Consequently, we delivered the siRNAs to HCFs and subsequently measured circSLC8a1 and SLC8a1 levels using real-time PCR and Western blotting. Consistently, the silencing of SLC8a1 resulted in decreased SLC8a1 mRNA and protein levels, whereas the silencing of circSLC8a1 led to reduced levels of both circSLC8a1 (*Figure 1K*) and SLC8a1-604 (*Figure 1L*). These findings provide confirmation that endogenous circSLC8a1 is responsible for the synthesis of the 75 kDa protein, SLC8a1-604.

**Figure 2:**
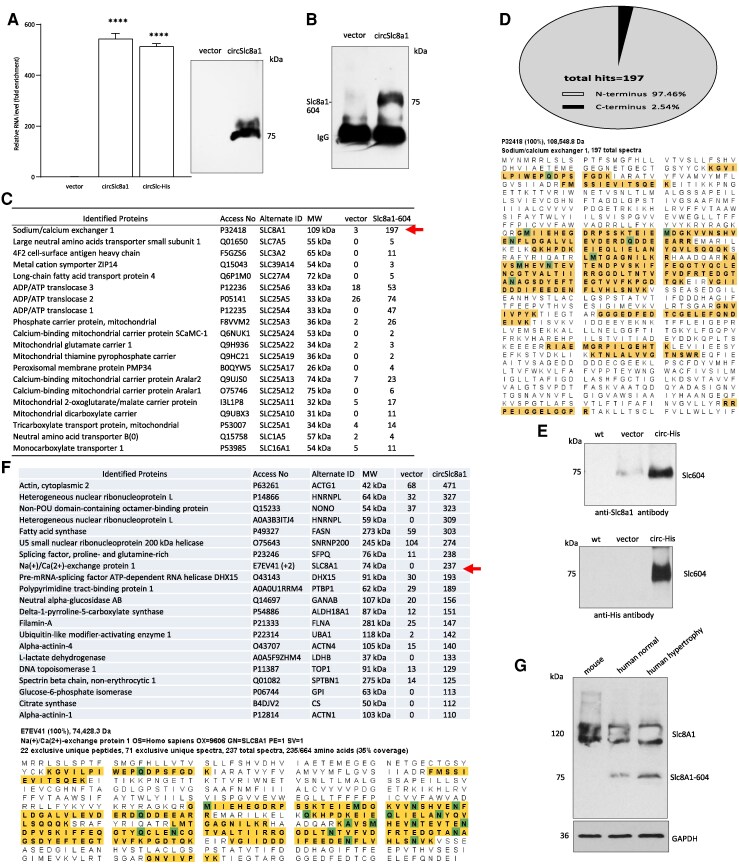
Characterization of SLC8a1-604. (*A*) Left, Addition of a His-tag did not affect the circularization of circSLC8a1. ****P < 0.0001 vs. vector control (*n = 6*), analysed by a one-way ANOVA followed by a Bonferroni post hoc test. Right, the cell lysate prepared from 293T cells transfected with the circSLC-His construct was subjected to His-column purification showing a specific band at the expected size. (*B*) The cell lysate prepared from 293T cells transfected with the circSLC8a1 construct was subjected to anti-SLC8a1 antibody precipitation, followed by Western blotting. A clear band at the expected size was detected in the cells transfected with circSLC8a1. (*C*) Lysates prepared from 293T cells transfected with circSLC8a1 or the vector were incubated with anti-SLC8a1 antibody. Precipitated products were subjected to LC-MS/MS analysis. Total spectra counts were indicated as numbers. Significantly higher counts of SLC8a1 peptides were obtained in the circSLC8a1 group relative to the vector group (red arrow). (*D*) The MS-reported peptide sequences were searched against SLC8a1 (highlighted). In the circSLC8a1-transfected group, most of the peptides matched the N-terminus of SLC8a1. (*E*) His-column purified proteins were analysed by Western blot probed with anti-His antibody (upper) and antibody against the SLC8a1 N-terminal sequence (lower). (*F*) The His-pull-down proteins underwent Mass spectrometry analysis. Red arrow, identification of SLC8a1-604 (upper). The MS-reported peptide sequences were searched against SLC8a1 (highlighted). In the circSLC8a1-transfected group, all peptides matched to SLC8a1-604 sequence (lower). (*G*) Lysates were prepared from mouse heart, human heart tissue without or with hypertrophy, were subjected to Western blotting probed with anti-SLC8a1 antibody. SLC8a1-604 was detected in human heart tissues. Data in (*A*) were analysed by a one-way ANOVA followed by a Bonferroni post hoc test.

While all the above results provided strong evidence showing the translation potential of circSLC8a1, more direct evidence was obtained by inserting a His-tag into the circSLC8a1 (circSLC-His). After confirming that the insertion of the His-tag at this location did not affect circularization efficiency (*Figure 2A*, left), we precipitated SLC8a1-604His using a His-column and detected the precipitated product by anti-His antibody. A specific band was obtained (*Figure 2A*, right). Furthermore, we also precipitated SLC8a1-604 using the N-terminus antibody. The precipitated product was observed through a Western blot detected by the N-terminus antibody. Compared to the vector control, circSLC8a1 transfection followed by the precipitation showed a clear product (*Figure 2B*). The precipitated product was subjected to mass spectrometry (MS/MS) to confirm the existence of the translated peptide. In comparison to the vector control, transfection with circSLC8a1 led to the precipitation of proteins targeted by the antibody against SLC8a1, displaying the highest number of read-counts (*Figure 2C*). We analysed the recognized peptides in the MS/MS results and found that overexpression of circSLC8a1 plasmid showed significantly more exclusively recognized peptides at the N-terminus, whereas only three peptides with one spectrum hit each were detected from the C-terminus (*Figure 2D*). These results suggest that the antibody-precipitated proteins were mainly translated by circSLC8a1.

Protein translated by the His-containing construct was purified using the His-column and subjected to Western blot probed by antibodies against His-epitope and SLC8a1. While incubation with SLC8a1 antibody showed a major band of 75 kDa, incubation with His specific antibody also showed specifically the 75-kDa protein band for SLC8a1-604 (*Figure 2E*), which was translated by circSLC8a1. Some trace amount of endogenous SLC8a1-604 was detected in the vector-transfected cells. Finally, the protein precipitated using the His-column was subjected to MS/MS analysis. As shown in *Figure 2F* (upper), one of the top recognized proteins was SLC8a1, and it was indicated in the protein-size column that the protein was recognized as 75 kDa, the exact predicted size of our polypeptide SLC8a1-604. Taken together, our results showed that circSLC8a1 possessed translational potential and it was translated to a protein isoform SLC8a1-604. The peptide sequences reported in the mass spectrometry (MS) analysis exhibited a 100% match with the SLC8a1-604 sequence (*Figure 2F*, lower). To further substantiate the presence of the 75 kDa protein *in vivo*, Western blotting was conducted on mouse and human heart tissues using an anti-SLC8a1 antibody. The results revealed that the 75-kDa protein was exclusively detected in human heart tissues, with higher levels observed in hypertrophic tissues compared to normal heart tissue, whereas it was absent in mouse heart tissue (*Figure 2G*).

### 3.2 SLC8a1-604 protein isoform mediates decreased cardiac functions

**Figure 3:**
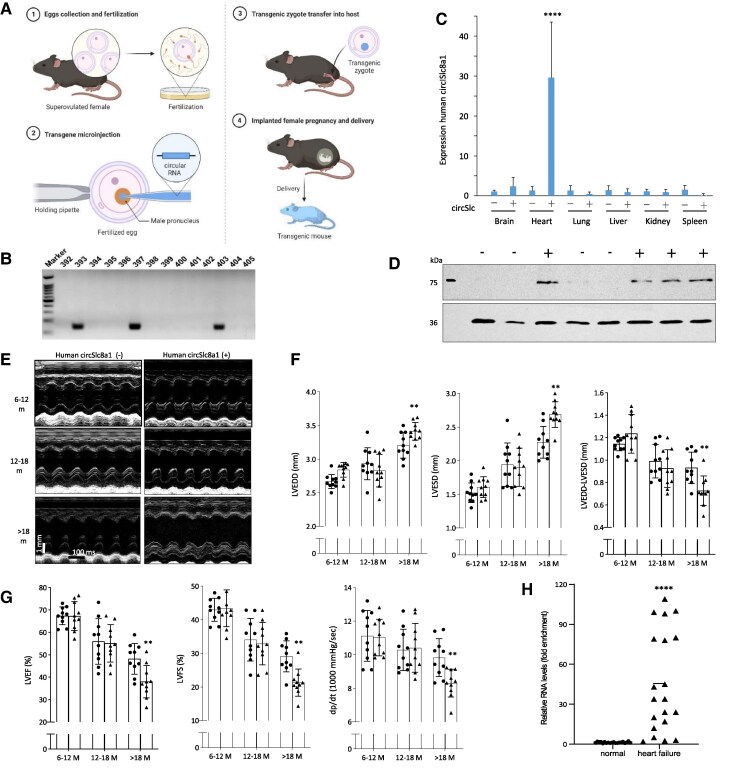
Decreased heart functions in the circSLC8a1 transgenic mice. (*A*) Diagrammatic representation of pronuclear microinjection procedure. (*B*) Breeders’ identification for the successful construction of the circSLC8a1-transgenic mouse line. (*C*) Tissues collected from the negative and positive transgenic mice were subjected to RNA extraction and RT-qPCR. Significantly higher levels of circSLC8a1 were observed in positive mice heart samples. *n* = 6, ****P < 0.0001 vs. vector control ±SD. (*D*) Heart samples collected from the transgenic mice were subjected to Western blotting. Four positive mice showed clear bands at the expected size. (*E*) Representative echocardiography M-mode images in old transgenic mice compared to the negative control mice. Positive mice older than 18 months developed cardiac remodelling characteristics. (*F*) Left, Increased left ventricular end-systolic diameter (LVESD) in the transgenic (TG) mice of age older than 18 months. Middle, Increased left ventricular end-diastolic diameter (LVEDD) in the transgenic (TG) mice of age older than 18 months. Right, Decreased subtraction of LVEDD by LVESD in the transgenic (TG) mice at the age older than 18 months. *n* = 10, **P < 0.01 vs. negative control ±SD. (*G*) Decreased left ventricular ejection fraction (LVEF, left) and left ventricular fractional shortening (LVFS, middle) were observed in the positive mice with ages older than 18 months. Right, Decreased left ventricular pressure (dp/dt) was observed in the positive mice with age older than 18 months. *n* = 10, **P < 0.01 vs. negative control ±SD. (*H*) Significantly increased levels of circSLC8a1 in the heart failure patients compared to the normal hearts. ****P < 0.0001 vs. normal ± SD. Data in c, f, g, and h were analysed by two-tailed unpaired Student’s *t*-test.

To examine the function of the circSLC8a1 translated peptide SLC8a1-604 *in vivo*, we established a transgenic mouse model expressing circSLC8a1 with a CMV promoter as per standard protocols (*Figure 3A*). The DNA fragment containing the promoter, the back-splicing unit, and the circSLC8a1 sequence was purified from the DNA gel for transgenic mouse line generation (see Supplementary material online, Figure  *S6*). We obtained three founder mouse lines after microinjection and observed similar levels of integration in the transgenic offspring (*Figure 3B*). One of them was used for further examination. Tissues from transgenic mice were collected and subjected to either RNA extraction followed by RT-qPCR and Western blotting. CircSLC8a1 was detected at a significantly higher level in the positive mouse hearts (*Figure 3C*). Specific bands at the designated sizes were obtained in the positive mouse hearts (*Figure 3D*).

Notable phenotypic changes were observed in the circSLC8a1-transgenic mice compared to the litter-matched negative counterparts. Echocardiography assessments were conducted on the transgenic mice at later developmental stages. The older circSLC8a1-transgenic mice exhibited characteristics of cardiac hypertrophy and remodelling (*Figure 3E*). Additionally, mice over 18 months of age displayed a significant decrease in ventricular contractile ability, as evidenced by increased left ventricular end-systolic diameter (LVESD), increased left ventricular end-diastolic diameter (LVEDD), and a decrease in the difference between LVEDD and LVESD (*Figure 3F*). Furthermore, 18-month-old circSLC8a1-transgenic mice (Tg mice) demonstrated a significant reduction in left ventricular ejection fraction (LVEF), left ventricular fractional shortening (LVFS), and left ventricular pressure (dp/dt) when compared to the negative control (*Figure 3G*). Taken together, these measurements indicated a substantial decline in heart function among the circSLC8a1-transgenic mice. To validate these findings, circSLC8a1 levels were assessed in human heart specimens. A total of 20 hearts without detectable cardiovascular diseases were collected from individuals who died from non-cardiac causes, while 20 heart failure samples were collected for circSLC8a1 measurements (see Supplementary material online, *Table S1*). The results showed significantly higher levels of circSLC8a1 in patients with heart failure compared to the normal donated heart specimens (*Figure 3H*). These findings further support the role of circSLC8a1 in the progression of cardiac remodelling and suggest the potential of circSLC8a1 as a therapeutic target.

**Figure 4:**
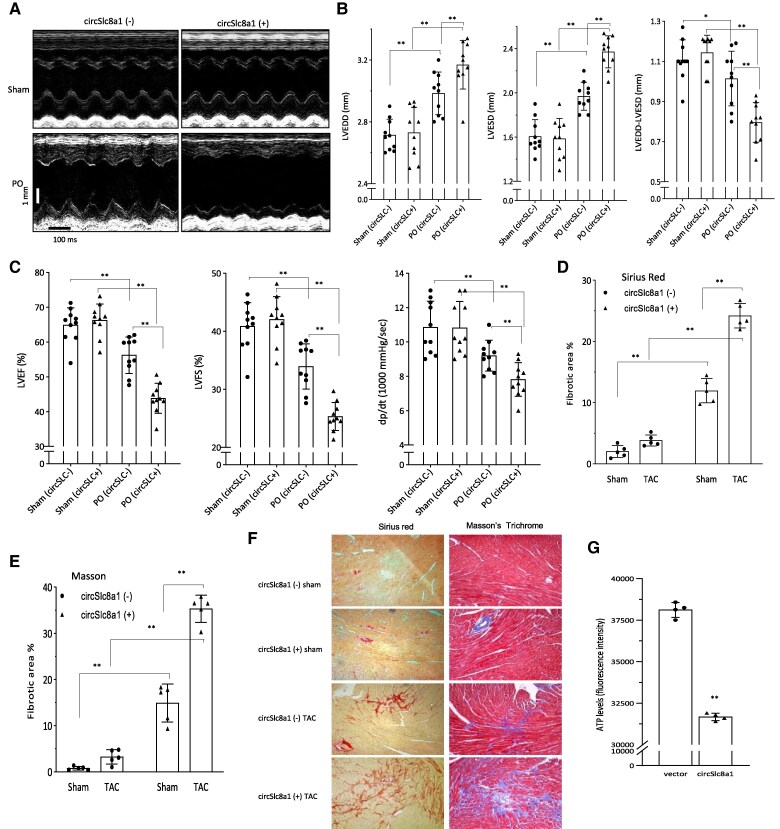
CircSLC8a1 decreased cardiac functions and increased fibrosis in the pressure-overload mice. (*A*) Representative M-mode images from the transgenic mice who underwent TAC model. (*B*) Echocardiography analysis showed increased LVEDD and LVESD but decreased subtraction between them in the pressure-overload mice compared to the sham control. Positive mice showed decreased heart functions compared to the negative control. *n* = 10, *P < 0.05, **P < 0.01 vs. negative or sham controls ± SD. (*C*) Haemodynamic analysis showing decreased LVEF, LVFS, and left ventricular pressure in the pressure-overload-negative mice compared to the sham control. Positive mice showed a decrease in haemodynamic analysis compared to the negative control. *n* = 10, *P < 0.05, **P < 0.01 vs. negative or sham control ± SD. (*D–E*) Statistical analysis of the Sirius Red (*D*) and Masson’s Trichrome (*E*) staining showing increased fibrosis in the positive mice following TAC inducement. *n* = 6, **P < 0.01 vs. negative or sham control ± SD. (*F*) Representative images of Sirius Red and Masson Trichrome staining. While mice with the TAC model displayed higher levels of collagen deposition compared to the sham group, the positive transgenic mice displayed more collagen deposition. (*G*) ATP levels were significantly decreased by circSLC8a1 in the heart tissues of the transgenic mice. *n* = 4, ** *P* < 0.01 vs. negative control ± SD. Data in b, c, d, and e were analysed by one-way ANOVA followed by Bonferroni post hoc test. Data in g were analysed by two-tailed unpaired Student’s *t*-test.

To investigate the impact of circSLC8a1 on heart function, we performed transverse aortic constriction (TAC) surgery in 6-month-old mice to induce PO, anticipating that it would enhance the effect of circSLC8a1. Notably, the circSLC8a1-transgenic mice exhibited a significant decrease in heart function compared to the negative mice after 4 weeks of TAC (*Figure 4A*). This reduction in heart function was evident through a decrease in the subtraction of LVEDD by LVESD, as well as reductions in LVEF, LVFS, and dp/dt (*Figure 4B* and *C*). These results further support the notion that circSLC8a1 expression facilitates cardiac remodelling.

Given that cardiac remodelling often leads to cardiac fibrosis, we conducted Sirius Red and Masson's Trichrome staining to examine cardiac tissues in the circSLC8a1-transgenic and negative control mice with or without pressure overload. Following tissue collection, heart sectioning, and staining, we observed significantly increased levels of fibrosis staining in the circSLC8a1(+) mice with pressure overload (*Figure 4D–F*). Furthermore, considering that mitochondria play a critical role in maintaining cardiac function, we measured the ATP content within the mitochondria of heart tissues from the circSLC8a1-transgenic mice. Interestingly, we observed a significant decrease in ATP content in the mitochondria of the circSLC8a1-transgenic mice (*Figure 4G*). This suggests that SLC8a1-604 is crucial for regulating mitochondrial function, particularly in relation to ATP production.

To further investigate the effects of circSLC8a1, we isolated primary cardiomyocytes from the transgenic and negative control mice and detected a decreased survival rate in the circSLC8a1(+) mice compared to the negative controls (see Supplementary material online, Figure  *S7*). Transfected HL-1 cell lines with either vector control or circSLC8a1 plasmids showed similar results in survival (see Supplementary material online, Figure  *S8*).

### 3.3 The impaired heart functions were due to the translated peptide

**Figure 5:**
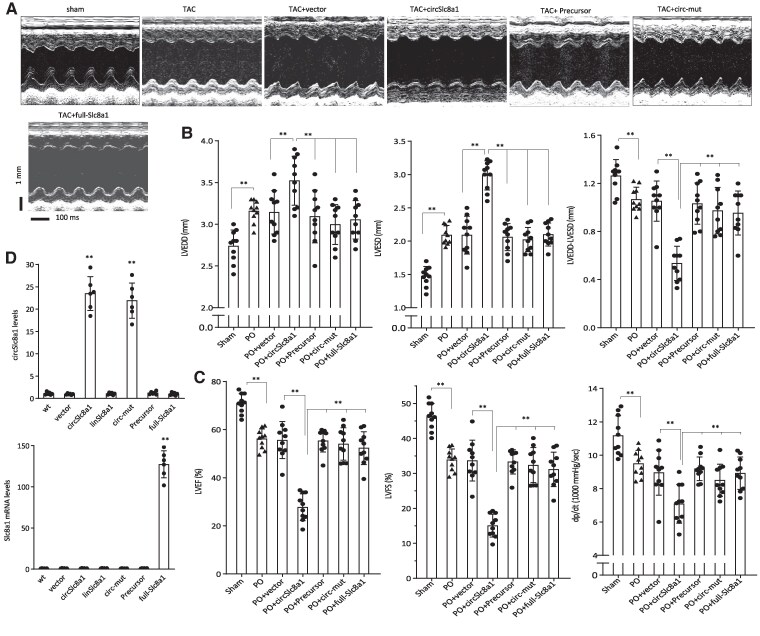
SLC8a1-604 was an essential mediator exerting circSLC8a1 functions. (*A*) Representative M-mode images of different circSLC8a1 plasmids delivery following TAC model inducement. Sham, no TAC mice; TAC, TAC mice without plasmid injection; TAC + vector, TAC mice injected with vector; TAC + circSLC8a1, TAC mice injected with circSLC8a1 plasmid; TAC + Precursor, TAC mice injected with a plasmid expressing circSLC8a1 precursor; TAC + circ-mut, TAC mice injected with a mutation circSLC8a1 that did not encode protein; TAC + full-SLC8a1, TAC mice injected with a plasmid expressing the full-length SLC8a1. (*B*) Pressure-overload mice displayed decreased heart functions compared to the sham control. CircSLC8a1 plasmid that has the translation potential further increased LVEDD (left), LVESD (middle), and decreased subtraction of LVEDD by LVESD (right). Other plasmids including precursor plasmid (Precursor), translation initiation mutated plasmid (cir-mut), and full-length linear plasmid (SLC8a1 linear) did not display any significant change in heart function following TAC. *n* = 10, **P < 0.01 vs. vector or sham control ± SD. (*C*) PO mice showed decreased haemodynamic measurements compared with the sham control. Delivery of circSLC8a1 that translated SLC8a1-604 further decreased heart functions as displayed by decreased LVEF (left), LVSF (middle), and dp/dt (right). Precusor plasmid (Precursor), translation initiation site mutation plasmid (cir-mut), and full-length plasmid (SLC8a1 linear) did not show significant differences compared with the vector control. *n* = 10, **P < 0.01 vs. vector or sham control ± SD. (*D*) 293T cells were transfected without or with vector, circSLC8a1, linSLC8a1, circ-mut, Precursor, and full-SLC8a1 followed by real-time PCR. Transfection with circSLC8a1 and circ-mut produced PCR product amplified by the circSLC8a1 primers (upper). Transfection with full-SLC8a1 produced full-length SLC8a1 mRNA (lower). Data in b, c, and d were analysed by one-way ANOVA followed by a Bonferroni post hoc test.

A previous study reported that the mouse circSLC8a1 was not likely to be translated.^25^ Since our results showed translation of human circSLC8a1 in the circSLC8a1-transgenic mice producing a new protein isoform SLC8a1-604, we examined the functions of SLC8a1-604 in detail. To test whether circularization of human circSLC8a1 was essential for circSLC8a1 functioning, we mutated the 5’intron, a fragment essential for backsplicing, resulting in the abolishment of circSLC8a1 circularization producing a linear fragment of SLC8a1-1829 nt (Precursor). To test whether the translation was essential for circSLC8a1 functioning, we generated a point mutation in the circSLC8a1 expression construct abolishing circSLC8a1 translation (circ-mut). In the TAC pressure-overload model, we delivered circSLC8a1 plasmid along with the Precursor, circ-mut, a SLC8a1 full-length linear plasmid (full-SLC8a1), and a vector control to the WT C57 mice. We found that only circSLC8a1 showed the most significant decrease in heart functions compared with the other delivered plasmids (*Figure 5A*). The reduced heart functions were also shown by the decrease in the subtraction of LVEDD by LVESD (*Figure 5B*), and decrease in LVEF, LVFS, and dp/dt (*Figure 5C*). These results suggest that circularization and circSLC8a1 translation are essential for circSLC8a1 functioning. The observed effects of circSLC8a1 on reducing cardiac functions most likely came from the translated peptides, rather than the effect of the circRNA itself or its potential combination with other binding partners. Nevertheless, the full-length SLC8a1 did not appear to have a similar function as SLC8a1-604. The expression of these constructs was verified using 293T cells (*Figure 5D*), known for their high efficiency in gene transfection and expression.

**Figure 6:**
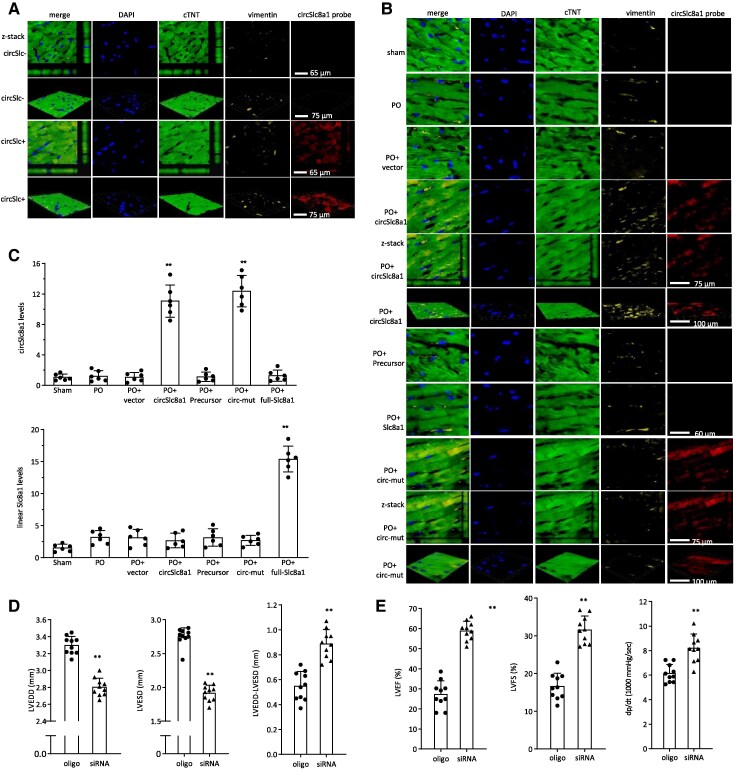
Expression and localization of ectopic circSLC8a1. (*A*) Representative z-stack hybridization and immunofluorescence images, including xy, xz, and yz projections and orthogonal views, depict circSLC8a1 (red), 4',6-diamidino-2-phenylindole (DAPI) (blue), cardiac troponin T (cTNT, green), and vimentin (yellow) in wild-type (wt) and circSLC8a1-transgenic mouse heart tissues. (*B*) Representative z-stack hybridization and immunofluorescence images illustrate circSLC8a1 (red), Dapi (blue), cardiac troponin T (cTNT, green) and vimentin (yellow) in PO mouse heat tissues injected with circSLC8a1 and circ-mut plasmids. (*C*) RNAs were extracted from the aforementioned treated mouse heart tissues and subjected to RT-PCR. Expression of circSLC8a1 (upper) and linear SLC8a1 mRNA (lower) levels in heart tissues was confirmed by real-time PCR. *n* = 6, **P < 0.01 vs. PO + vector ±SD. (*D*) Decreased LVEDD (left), LVESD (middle) and increased subtraction of LVEDD by LVESD (right) were observed in si-circSLC8a1 mice compared to the oligo control group. *n* = 10, **P < 0.01 vs. oligo control ±SD. (*E*) Increased LVEF (left), LVSF (middle), and dp/dt (right) were observed in the siRNA delivery group compared to the oligo control group. *n* = 10, **P < 0.01 vs. oligo control ±SD. Data in c were analysed by one-way ANOVA followed by a Bonferroni post hoc test. Data in d 4 and e were analysed by two-tailed unpaired Student’s *t*-test.

Successful delivery and expression of the plasmids were validated by immunofluorescent in situ hybridization. Typical z-stack immunofluorescent images were acquired. In the heart tissues of circSLC8a1-transgenic and litter-matched negative mice, immunofluorescent images and z-stack confirmed the expression of circSLC8a1 in the transgenic mice. This expression overlapped with vimentin, used as a reference to identify cardiac fibroblasts, and cardiac troponin T (cTNT), serving as a reference for cardiomyocyte localization (*Figure 6A*). In the PO mice, delivery of circSLC8a1 resulted in increased levels of circSLC8a1 staining (*Figure 6B*). However, the delivery of circSLC8a1 precursor and the full-length SLC8a1 constructs did not yield the same increase, whereas the delivery of the mutation construct (circ-mut) plasmid did elevate the levels of in situ hybridization staining (*Figure 6B*). Confirmation of successful plasmid delivery was further established through real-time PCR measurements (*Figure 6C*).

To validate the impact of circSLC8a1, we employed a siRNA approach to specifically target circSLC8a1. We designed siRNAs against circSLC8a1 and delivered them using nanoparticle conjugation to the circSLC8a1-transgenic mice undergoing TAC-induced pressure overload. Notably, we observed that silencing circSLC8a1 effectively mitigated the detrimental effects observed on echocardiography in mice treated with nanoparticle-conjugated circSLC8a1-siRNAs (*Figure 6D* and *E*, Supplementary material online, Figure  *S9*). These findings provide additional evidence that targeted inhibition of circSLC8a1 can counteract the adverse cardiac phenotypes associated with pressure overload, as demonstrated by improved echocardiographic parameters.

### 3.4 Decreased expression of mitochondrial genes in the SLC8a1-604 mice

**Figure 7:**
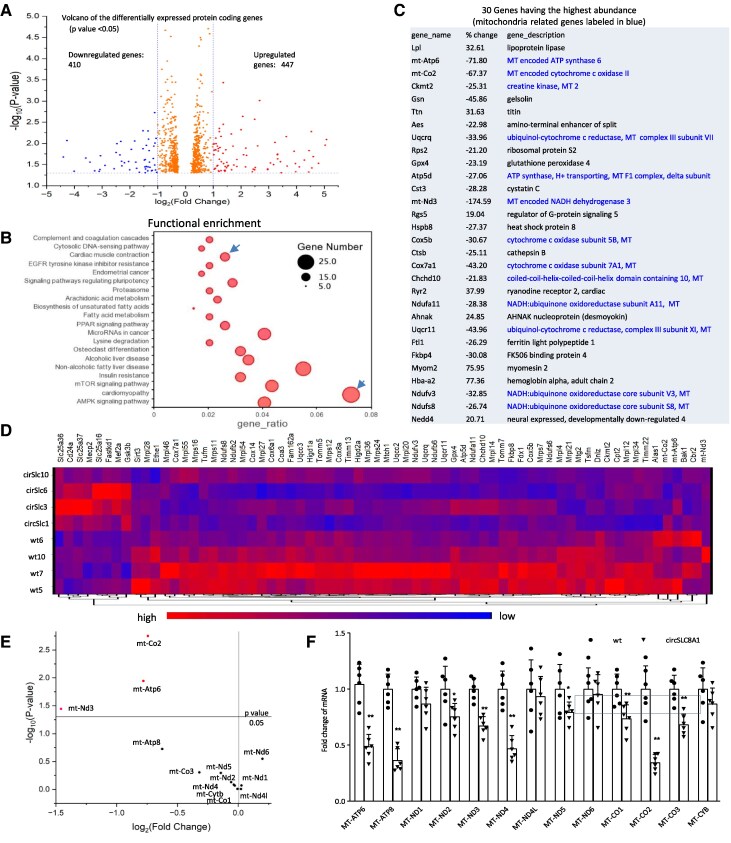
Sequencing and analysis of RNAs isolated from circSLC8a1 transgenic mice. (*A*) A volcano plot displays 25 278 transcripts detected by RNA sequencing, with 857 protein-coding genes showing differential expression at *P* values < 0.05. (*B*) Among the 857 differentially expressed genes, the top 20 functional enrichment pathways are listed, with cardiomyopathy being the highest. (*C*) The top 30 genes with the highest abundance, including 13 mitochondria-related genes, are listed. All of them exhibit downregulation in circSLC8a1-transgenic mice. (*D*) Of the 857 genes, 68 are related to mitochondrial functions. The heatmap illustrates that 60 of them are downregulated, while 8 are upregulated. (*E*) A volcano plot focuses on the 13 protein-coding genes encoded by the mitochondrial genome, with 10 downregulated and 3 upregulated. (*F*) Mouse heart tissues were lysed and subjected to RT-PCR. Graphs display the mRNA fold change of the 13 mitochondrial genes in circSLC8a1-transgenic mice compared to wt mice. *n* = 6, **P < 0.01 vs. wt ± SD, analysed by two-tailed unpaired Student’s *t*-test.

To uncover the molecular mechanism underlying the effect of SLC8a1-604, we conducted mRNA sequencing of cardiac mRNAs. All samples had approximately 6-G of clean reads (see Supplementary material online, *Table S2*), with satisfactory distribution in terms of mapping to reference genes (see Supplementary material online, *Table S3*), gene expression (see Supplementary material online, Figure  *S10*), and Pearson correlation (see Supplementary material online, *Table S4*). Transcriptome analysis unveiled 937 genes with differential expression, meeting the criteria of a *P*-value smaller than 0.05 and a 2-fold cut-off (*Figure 7A*). Our Kyoto Encyclopedia of Genes and Genomes (KEGG) analysis revealed the highest proportion of genes were associated with cardiomyopathy (*Figure 7B*). Enrichment analysis highlighted a significant involvement in mitochondrial and energy-related pathways (see Supplementary material online, Figure  *S11*). Notably, among the top 30 genes with the highest abundance, 13 were linked to mitochondria (*Figure 7C*). These results suggested the role of mitochondria in mediating SLC8a1 functions, consistent with our observation that ATP production decreased in the circSLC8a1-transgenic mice (*Figure 4G*).

Subsequently, we investigated the expression of mitochondria-associated genes and identified 68 genes with differential expression (*Figure 7D*). Of these, 60 were downregulated, and 8 were upregulated (*Figure 7D*). Additionally, we examined the 13 genes encoded by the mitochondrial genome and found that 10 of them were downregulated, with 3 showing upregulation (*Figure 7E*). Notably, at a statistically significant level (*P* < 0.05), only three genes were significantly downregulated, indicating that the regulation mediated by SLC8a1-604 likely occurs within the mitochondria. To validate these findings, we designed primer pairs for all 13 genes expressed in the mitochondria and conducted real-time PCR on heart tissues from the SLC8a1-604 transgenic mice. The results clearly demonstrated significant downregulation of 9 genes in the SLC8a1-604 heart tissues compared to the wild-type controls (*Figure 7F*).

### 3.5 Complex formation of SLC8a1-604, POLRMT, and TIMM50

**Figure 8:**
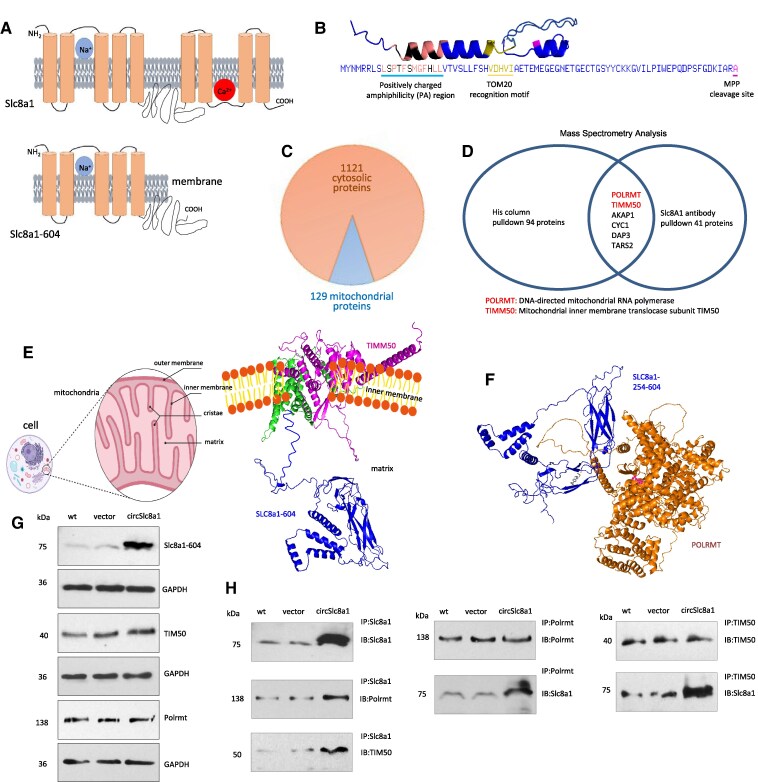
Interaction of SLC8a1-604 with TIMM50 and POLRMT. (*A*) The structure of SLC8a1-604 and SLC8a1 proteins is shown. (*B*) The N-terminal 78 amino acids of SLC8a1-604, highlighting the Positively Charged Amphiphilicity (PA) region, TOMM20 recognition motif, and MPP cleavage site. (*C*) Mass spectrometry analysis revealed that the N-terminal SLC8a1 antibody-precipitated 1250 proteins, including 1121 cytoplasmic proteins and 129 mitochondria-associated proteins. (*D*) The proteins precipitated by His column (94 proteins) and anti-SLC8a1 antibody (41 proteins) were merged. Six of them were common, including DNA-directed mitochondrial RNA polymerase (POLRMT) and mitochondrial inner membrane translocase subunit TIM50 (TIMM50). (*E*) Left, A diagram shows mitochondrial structure. Right, Interaction of SLC8a1-604 with TIMM50 derived by computational algorithm. (*F*) Interaction of SLC8a1-604 with POLRMT derived by computational algorithm. (*G*) 293T cells transfected with or without circSLC8a1 and control vector were subjected to Western blotting probed with antibodies against SLC8a1 (N-terminal), TIMM50, and POLRMT. Transfection with circSLC8a1 did not affect TIMM50 and POLRMT expression. (*H*) Cell lysates were immunoprecipitated with antibodies against SLC8a1, POLRMT, and TIMM50, respectively, followed by Western blotting. Precipitation of SLC8a1-604 pulled down POLRMT and TIMM50 (left). Precipitation of POLRMT (middle) or TIMM50 (right) pulled down SLC8a1-604.

The SLC8a1-604 peptide contains a signal sequence (amino acids 1-35), 5 transmembrane domains (219 amino acids, AA 36-254), and a free internal C-terminal fragment (350 amino acids, AA 255-604). This internal C-terminal fragment hangs inside the cellular membrane. In the full-length SLC8a1 protein, this 350-amino acid peptide forms a loop to connect with the C-terminal fragment (*Figure 8A*).

Computational analysis predicted the potential interaction of SLC8a1-604 with the mitochondrial membrane (*Figure 8B*). Given that SLC8a1 is an ion channel translocated to the cell membrane and SLC8a1-604 contains five transmembrane motifs, there is a possibility that SLC8a1 could translocate to both the cell surface and the mitochondrial membrane. Our hypothesis was that this free-hanging peptide might interact with protein partners different from the loop region, resulting in distinct functionality compared to the full-length SLC8a1 protein. To test this hypothesis, we utilized an antibody recognizing the N-terminus of SLC8a1 protein to precipitate SLC8a1-604. The proteins interacting with SLC8a1-604 were then isolated by the antibody and subjected to LC-MS/MS analysis. This analysis revealed that a substantial number of mitochondrial proteins were pulled down by the antibody precipitating SLC8a1-604, in contrast to the vector control (see Supplementary material online, *Table S5*). Importantly, the mitochondrial proteins precipitated by SLC8a1-604 exhibited significantly higher read-counts relative to the control.

To validate these findings, we conducted another pull-down assay using a His-column to bind the His-tag-containing SLC8a1-604. The His-column also successfully pulled down numerous mitochondrial proteins, as indicated by the MS assay (see Supplementary material online, *Table S6*). By combining the lists of proteins from Supplementary material online, *Table S5* and *S6*, we identified six common proteins. These proteins were likely to specifically interact with SLC8a1-604 (*Figure 8D*). Among the common proteins, one was the mitochondrial inner membrane protein TIMM50 (mitochondrial import inner membrane translocase subunit TIM50), and another was the DNA-directed RNA polymerase, mitochondrial (POLRMT), which is responsible for mitochondrial gene expression and providing RNA primers for mitochondrial gene replication initiation.

Computational analysis suggested that the five transmembrane motifs of SLC8a1-604 could interact with TIMM50 (*Figure 8E*). Additionally, it indicated that the free C-terminus of SLC8a1-604 could interact with POLRMT (*Figure 8F*). To experimentally validate these interactions, we conducted co-immunoprecipitation assays in 293T cells transfected with or without circSLC8a1 and the control vector. Subsequent Western blotting confirmed increased levels of SLC8a1-604 following circSLC8a1 transfection (*Figure 8G*). The co-immunoprecipitation assay revealed that precipitation of SLC8a1-604 co-precipitated both TIMM50 and POLRMT (*Figure 8H*, left). Precipitation of POLRMT also co-precipitated SLC8a1-604 (*Figure 8H*, middle), while precipitation of TIMM50 co-precipitated SLC8a1-604 (*Figure 8H*, right).

**Figure 9:**
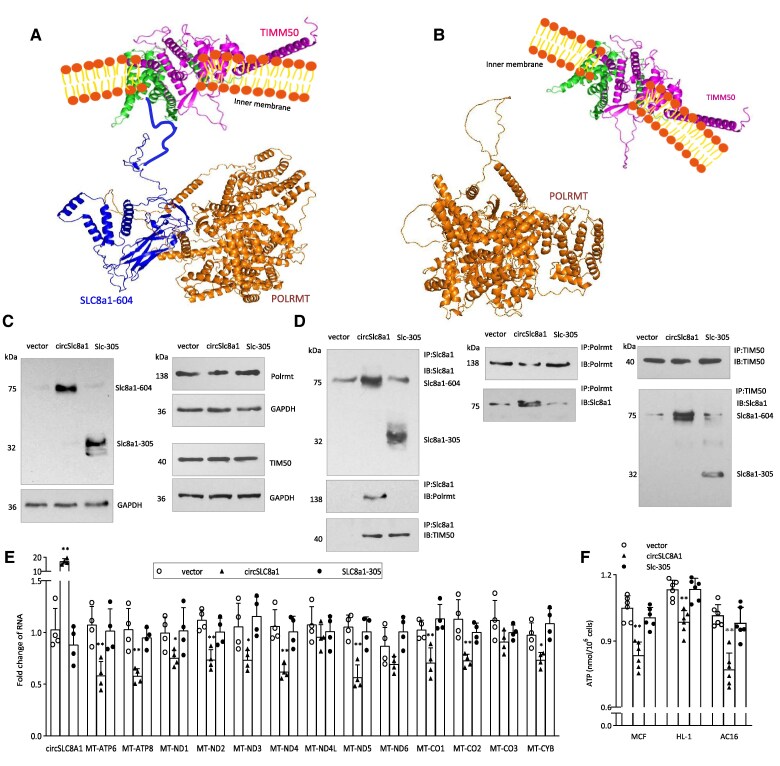
Truncation of the SLC8a1-254-604 abolished its interaction with POLRMT leading to a reverse effect of SLC8a1-604 on mitochondrial gene expression and ATP production. (*A*) Interaction of SLC8a1-604 with POLRMT and TIMM50 derived by computational algorithm. (*B*) Truncation of the SLC8a1-254-604 abolished its interaction with POLRMT according to computational algorithm. (*C*) 293T cells were transfected with control vector, circSLC8a1 and SLC8a1-305, and subjected to Western blotting with antibodies against SLC8a1, POLRMT, and TIMM50, confirming the expression of SLC8a1-604 and SLC8a1-305. (*D*) The lysates were immunoprecipitated with antibodies against SLC8a1, POLRMT, and TIMM50, respectively, followed by Western blotting. Precipitation of SLC8a1-305 pulled down TIMM50 but not POLRMT (left). Precipitation of POLRMT pulled down SLC8a1-604 but not SLC8a1-305 (middle). Precipitation of TIMM50 pulled down both SLC8a1-604 and SLC8a1-305 (right). (*E*) AC16 cells were transiently transfected with control vector, circSLC8a1 or SLC8a1-305, and cultured in 50 µM H_2_O_2_ for 24 h. RT-PCR analysis showed that the expression of circSLC8a1 but not SLC8a1-305, repressed the mRNA levels of 13 mitochondrial genes. *n* = 4, **P < 0.01 vs. wt ± SD. (*F*) Mouse cardiac fibroblasts (MCF), HL-1, and AC16 cells were transiently transfected with control vector, circSLC8a1 or SLC8a1-305, and cultured in 50 µM H_2_O_2_ for 24 h. ATP levels in cell lysates were analysed using an ATP Assay Kit. Expression of circSLC8a1, but not SLC8a1-305, suppressed ATP levels. *n* = 6, **P < 0.01 vs. wt ± SD.

Our results strongly suggested that SLC8a1-604, POLRMT, and TIMM50 could form a complex. Indeed, computational analysis revealed that these three proteins form a complex associated with the mitochondrial inner membrane (*Figure 9A*). We hypothesized that this complex formation might inhibit POLRMT's function in mitochondrial gene transcription. To test this, we examined whether the C-terminal fragment of SLC8a1-604 was responsible for the interaction with POLRMT (*Figure 9B*). We generated a mutation construct, SLC8a1-305, by truncating the C-terminal fragment of SLC8a1-604 (see Supplementary material online, Figure  *S4*). The cells were transfected with circSLC8a1 and SLC8a1-305, along with the control vector. Following transfection, cells were subjected to Western blotting. Transfection with SLC8a1-305 resulted in the production of a 32-kDa protein band (*Figure 9C*). To further substantiate these findings, we performed co-immunoprecipitation experiments using antibodies against SLC8a1, POLRMT, and TIMM50, respectively. Precipitation of SLC8a1-305 co-precipitated TIMM50 but not POLRMT (*Figure 9D*, left). However, precipitation of POLRMT no longer co-precipitated SLC8a1-305 (*Figure 9D*, middle), while precipitation of TIMM50 still successfully pulled down both SLC8a1-604 and SLC8a1-305 (*Figure 9D*, right).

To validate the crucial role of this interaction in downregulation of mitochondrial gene expression, we introduced the mutant construct into the human cell line 293T cells and assessed mitochondrial gene levels. Notably, we observed a significant downregulation of mitochondrial genes in cells transfected with circSLC8a1. However, this downregulation was not evident in cells transfected with the truncated version (*Figure 9E*). In functional assays, we found that transfection with circSLC8a1 led to a decrease in ATP production. In contrast, transfection with the mutant construct abolished this effect (*Figure 9F*). These results provide conclusive evidence that the interaction between SLC8a1-604 and POLRMT plays an essential role in the regulation of mitochondrial genome expression and ATP production.

## 4. Discussion

Abnormal elevation in circRNA levels has been closely linked to the onset and progression of cardiovascular diseases.^39–44^ A wide range of circRNAs has been demonstrated to play critical roles in various cardiovascular diseases, including cardiomyopathy, myocardial infarction, and artery diseases.^26,45–47^ These circRNAs have been recognized as potential therapeutic targets and valuable biomarkers for cardiovascular diseases.^47–52^ Therefore, it is of great importance to investigate the roles of circRNAs in the development of cardiovascular diseases. Recently, some other non-coding RNAs, such as miRNAs and lncRNAs, have been successfully applied in RNA therapy in cardiovascular diseases.^53^ However, the precise mechanisms through which circRNAs can be a target in RNA therapy are still the subject of ongoing investigation.

Our computational analysis suggests that human circSLC8a1 may be translated to a polypeptide with 604 amino acids (SLC8a1-604). Structural analysis showed that this translated protein contains five transmembrane domains and a large internal C-terminal fragment. There might be a couple of potential mechanisms for circSLC8a1 to exert its functions: (i) the circSLC8a1 translated protein may form a channel allowing Na^+^ to enter the cells, (ii) the free internal C-terminal fragment may bind to other proteins and mediate circSLC8a1 function, (iii) the circRNA circSLC8a1 may bind to signalling molecules and regulate cell activities, or (iv) there are several reports showing that circSLC8a1 can function as a miRNA sponge.^54–56^ In this study, we found that human circSLC8a1 translated to a peptide and such peptide is functional in mediating the progression of cardiac pressure-overload remodelling. Following *in vivo* functional comparison between the mice delivered with construct that translated peptide and constructs that do not form a circRNA or mutated translation initiation site abolishing translation, we found that the functions observed in echocardiography were mainly due to that translated peptide but not the circRNA itself. Furthermore, we found that the translated protein SLC8a1-604 did not mainly locate on the cell membrane as its parental gene. Instead, it translocated into the mitochondria where it modulated mitochondrial activities.

Our result showing translation of human circSLC8a1 is consistent with previous reports that human circSLC8a1 had the potential for protein-coding activity.^24^ While we analysed the sequence of human circSLC8a1, we found a stop codon starting at the nucleotide 5 beyond the junction of the circular RNA (nucleotides 5–7). As a result, only two unique amino acids were present in SLC8a1-604 as compared with the parental SLC8a1 protein, generating a protein with 604 amino acids. These two unique amino acids were neither long enough to be detected by the MS method, nor sufficient to be used to generate antibodies. Nevertheless, we used all other possible methods to confirm the translation of SLC8a1-604 by circSLC8a1. For instance, ribosome RNA binding assay showed that circSLC8a1 interacted with ribosome that was abolished by puromycin treatment. MS assay detected many more N-terminal peptides in the circSLC8a1-transfected cells. Transfection with the circSLC8a1 expression construct produced a unique 75 kDa protein band, similar to the pcDNA3.1 construct expressing a linear cDNA that could be translated to SLC8a1-604. Insertion of the His-tag into the circSLC8a1 expression construct showed a His-containing protein band of 75 kDa, similar to the pcDNA3.1 construct expressing the same amino acid sequence. Functionally, all of these constructs expressing SLC8a1-604 showed similar results. Site-directed mutagenesis blocked SLC8a1-604 translation resulting in functional abolishment of human circSLC8a1. Inhibition of circSLC8a1 circularization also abolished circSLC8a1 function. We thus concluded that human circSLC8a1 can be translated to produce SLC8a1-604 that is essential in mediating circSLC8a1 functions. On the other hand, mouse circSLC8a1 does not contain a stop codon in its junction sequence (see Supplementary material online, Figure  *S12A*, upper). Consequently, no mouse circSLC8a1-coding protein with 604 amino acids has been detected^25^ (see Supplementary material online, Figure  *S12B*, left). Nevertheless, it remains possible that mouse circSLC8a1 could be translated into large proteins through the rolling translational mechanism unique to circRNA translation.^10^ If this occurs, the situation becomes more complex due to the large size of circSLC8a1 and the presence of glycosylation sites in the potential encoded amino acid sequence. These factors could result in a glycosylated protein too large to be detected via Western blot. To confirm this, we engineered a stop codon at the end of the mouse circSLC8a1 (see Supplementary material online, Figure  *S12A*, lower). Transfection of the construct containing the stop codon allowed the detection of a protein band at the expected location (see Supplementary material online, Figure  *S12S*b, right).

Mitochondria possess critical physiological roles in maintaining and regulating cardiovascular systems. They play crucial roles in biological metabolic pathways and mediating intracellular Ca^2+^ fluxes. The malfunction of mitochondria has been stated to be involved in the pathogenesis of different cardiovascular diseases including hypertension, arrhythmias, myocardial infarction, and multiple cardiomyopathies of various aetiologies. Following damage to mitochondrial functions, a series of inflammatory responses and regulated cell death in cardiomyocytes would happen and further contribute to the pathogenesis and progression of cardiovascular diseases. In this study, we reveal that the protein translated from circSLC8a1 exerts its function by regulating the mitochondrial activity to some extent, thereby affecting the ATP synthesis. The translocation of SLC8a1-604 to mitochondria blocked the physiological activity of the mitochondrial proteins.

While the current study provides evidence of the existence and functionality of the SLC8a1-604 translated peptide, further research is required to fully elucidate the underlying mechanisms. This will be a crucial direction for future studies. For instance, it remains unclear why the full-length SLC8a1 is translocated to the cell surface membrane while the small isoform is translocated to the mitochondria. Notably, the full-length SLC8a1 comprises nine transmembrane domains, whereas SLC8a1-604 contains only five transmembrane domains, along with a C-terminal fragment responsible for binding to the mitochondrial protein POLRMT. This interaction may have a significant role in the mitochondrial targeting of SLC8a1-604, and further investigations are warranted to better understand this process.

In the mass spectrometry results, there were also structural proteins that got pulled down by SLC8a1-604 which may affect heart functions through regulating the contraction of myocytes. We do not eliminate the possibility that SLC8a1-604 generates functions through a more complex networking system, and regulating mitochondria may be one of the mechanisms. Further detailed analysis should be performed to trace the exact localization and translocation of this translated peptide. This includes using His-tag antibody for immunofluorescent staining and co-staining with subcellular markers (e.g. voltage-dependent anion channel or VDAC for mitochondria). Further functional analysis at both the cellular and subcellular levels should also be conducted, encompassing techniques such as Ca^2+^ imaging and measurements of myocyte contraction rates.

**Translational perspective**

This proof-of-concept study may lay the foundation for potential clinical applications in circular RNA therapy. Here, we provide evidence confirming the translation potential of circSLC8a1: circSLC8a1 translates a protein SLC8a1-604 that mediates the progression of cardiac pressure-overload remodelling. Silencing circSLC8a1 effectively mitigates the detrimental effects of circSLC8a1/SLC8a1-604 on mice treated with nanoparticle-conjugated siRNAs targeting circSLC8a1. Targeted inhibition of circSLC8a1 can counteract the adverse cardiac phenotypes associated with pressure overload. Our work contributes insights into the functional landscape of circSLC8a1, emphasizing its potential to encode the functional SLC8a1-604. The influence of SLC8a1-604 on heart function regulation highlights the clinical perspective of understanding circRNA-mediated translation events, offering new avenues for therapeutic interventions in heart failure.

## Supplementary Material

cvaf058_Supplementary_Data

## Data Availability

The data underlying this article will be shared on reasonable request to the corresponding author.
