# Supplementary material for: A new protein isoform encoded by human circular RNA circSLC8a1 contributes to cardiac remodelling
Source: Cardiovasc Res. 2025 Apr 24;121(6):882–99. doi: 10.1093/cvr/cvaf058 (PMC12160813; doi:10.1093/cvr/cvaf058)
Supplement: cvaf058_Supplementary_Data [file cvaf058_supplementary_data.pdf]

Contents of Supplementary Information

General Methods Pages 1-2
Supplementary Figures Pages 3-14
Supplementary Tables Pages 15-19

General Methods

Cardiac function assessment

Mice were anesthetized with 2% isoflurane inhalation to undergo transthoracic echocardiography. Transthoracic echocardiography was performed and analyzed in a blinded manner, using a Vevo 2100 high-resolution imaging system equipped with a 40-MHz transducer to record the m-mode image of the left ventricle and measure left ventricular end diastolic diameter (LVEDD), left ventricular end systolic diameter (LVESD), left ventricular ejection fraction (LVEF), left ventricular fractional shortening (LVFS) and dp/dt.

Subcellular fractionation

The subcellular fractions of the cells were isolated. In brief, the cultured cells or heart tissues were harvested and resuspended in 500 μl fractionation buffer (250mM sucrose, 20 mM HEPES pH 7.4, 10 mM KCl, 2 mM $MgCl_2$, 1 mM EDTA, 1 mM EGTA, and protease inhibitor cocktail). Then, the cells were homogenized by 10 passages through a 25-G needle using a 1 ml syringe and incubated on ice for 30 min. The heart tissues were homogenized in fractionation buffer using a Dounce homogenizer on ice. The nuclear pellet was collected by centrifugation at $720\times g$ for 5 min. The supernatant was centrifuged again at $10{,}000\times g$ for 15 min. The pellet containing mitochondria was collected followed by further wash with the fractionation buffer. The supernatant containing the cytosolic fraction was concentrated and purified with Millipore centrifugal filter units.

Western blotting

The protein expression levels were determined by Western blotting. In brief, the proteins were isolated from tissues or cells using RIPA buffer. Proteins in the lysates were separated by sodium dodecyl sulfate-polyacrylamide gel electrophoresis (SDS-PAGE). The separated proteins were transferred onto a nitrocellulose membrane in 1x Tris/glycine buffer containing 20% methanol at 80 V and 4 ° C for 2 h. The membranes were blocked in TBST buffer containing 5% (w/v) non-fat dry milk powder for 1 h followed by incubation with primary antibodies at 4 ° C overnight. The membranes were then washed and incubated with secondary antibodies at room temperature for 2 h. After washing, the bound antibodies were visualized with an ECL detection kit (Millipore). The antibodies against SLC8a1 (NCX1), TOMM40, TIMM50, ATPB were from ABclonal. Anti-GAPDH monoclonal antibody (Proteintech) was used to confirm equal loading of the proteins for each sample.

ATP assay

An ATP assay kit (Abcam #A22066) was used to measure the ATP levels in the heart tissues according to the manufacturer's instructions. Briefly, the cultured cells were resuspended and homogenized in ATP assay buffer followed by centrifugation at 13,000xg at 4 ° C for 5 min. The supernatant was collected and deproteinized by using 1 M perchloric acid (PCA). The heart tissues were homogenized in ice cold PCA with a Dounce homogenizer and kept on ice for 30-45 min. After centrifugation at 13,000xg at 4 ° C for 2 min, the supernatant was neutralized to pH 6.5-8 with KOH. Then, the supernatant, which was collected after another centrifugation, and the ATP standard were applied for fluorometric assay with the ATP probe, ATP converter and Developer Mix provided in the assay kit.

Histological staining

After harvested, the hearts were cut into two halves. The lower halves were fixed with 10% buffered formalin and embedded in paraffin, processed into 5 µm sections. Masson's trichrome staining and Sirius red staining were performed to evaluate cardiac fibrosis. Masson's trichrome stain kit (American Master Tech) was used for Masson's trichrome staining according to the manufacturer's instructions. For Sirius red staining, mouse heart sections were de-waxed and hydrated, followed by staining with Weigert's hematoxylin for 8 min. The sections were washed with running tap water, stained in 0.1% picrosirius red for 1 h, and washed with 0.1% acetic acid.

Fluorescence *in situ* Hybridization (FISH)

DNA oligo probes targeting circSLC8a1 and labeled with Alexa 488 or 546 were synthesized using the fluorescence PCR labeling kit from Biolynx. The probes underwent heat denature at 95° C for 2 minutes and were then chilled on ice prior to use. A labeled scramble sequence served as a control. To prepare the tissue sections, de-paraffinization was carried out with xylene, followed by dehydration using 75% ethanol, 95% ethanol, and 100% ethanol. The sections were pre-treated with hybridization solution (Ambion) at 56° C for 30 minutes. Subsequently, the slides were incubated with 50 nM fluorescence-labeled DNA oligo probes in hybridization buffer at 56° C for 4 hours, followed by sequential washes with saline-sodium citrate (SSC) buffers. After blocking with TBS containing 10% goat serum for30 minutes, the slides underwent additional immunofluorescence staining.

Immunofluorescence staining

Tissue sections underwent de-paraffinization using xylene and ethanol, followed by blocking with TBS containing 10% goat serum for 30 minutes. Following an overnight incubation with the primary antibody in TBS containing 10% goat serum, the slides were washed and then stained with goat anti-mouse or rabbit Alexa Fluor 488, 546, or 647 at room temperature for 2 hours. Fluorescent phalloidin 488 or phalloidin 546 was employed to stain F-actin, providing visualization of cell structure, while DAPI was used to stain DNA. Subsequently, images of the stained samples were captured using Nikon N-SIM S confocal laser scanning microscopy. The intensity of staining was analyzed using ImageJ.

Supplementary Figures

a

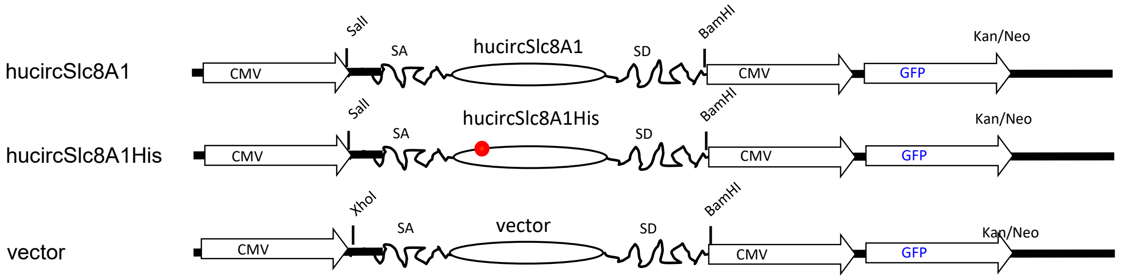

SA, splice acceptor; SD, splice donor

b

hucircSlc8A1

gtcgacacacttagccgtgttctttgcactttctgcatgtcccccgtctggcctggctgtccccagtggcttccccagtgtgacatggtgcatctctgccttacag
gttgtgacagttggaagtgtc**atg**tacaacatgcggcgattaagtctttcacccaccttttcaatgggatttcatctgttagttactgtgagtctcttattttcccatgtggaccatgtaattgct
gagacagaaatggaaggagaaggaaatgaaactggtgaatgtactggatcatattactgtaagaaaggggtgattttgcccatttgggaaccccaagacccttcttttggggacaaaatt
gctagagctactgtgtattttgtggccatggtctacatgtttcttggagtctctatcatagctgatcggttcatgtcctctatagaagtcatcacatctcaagaaaaagaaataaccataaag
aaacccaatggagagaccaccaagacaactgtgaggatctggaatgaaacagtttctaacctgaccttgatggccctgggatcttctgctcctgagattctcctttcagtaattgaagtgtg
tggccataacttcactgcaggagacctcggtcctagcaccatcgtgggaagtgctgcattcaatatgttcatcattattgcactctgtgtttatgtggtgcctgacggagagacaaggaaga
ttaagcatttgcgtgtcttctttgtgacagcagcctggagcatctttgcctacacctggctttacattattttgtctgtcatatctcctggtgttgtggaggtctgggaaggtttgcttactttctt
cttctttcccatctgtgttgtgttcgcttgggtagcggataggagacttctgttttacaagtatgtctacaagaggtatcgagctggcaagcagagggggatgattattgaacatgaaggag
acaggccatcttctaagactgaaattgaaatggacgggaaagtggtcaattctcatgttgaaaatttcttagatggtgctctggttctggaggtggatgagagggaccaagatgatgaag
aagctaggcgagaaatggctaggattctgaaggaacttaagcagaagcatccagataaagaaatagagcaattaatagaattagctaactaccaagtcctaagtcagcagcaaaaaa
gtagagcattttatcgcattcaagctactcgcctcatgactggagctggcaacattttaaagaggcatgcagctgaccaagcaaggaaggctgtcagcatgcacgaggtcaacactgaag
tgactgaaaatgaccctgttagtaagatcttctttgaacaagggacatatcagtgtctggagaactgtggtactgtggcccttaccattatccgcagaggtggtgatttgactaacactgtgt
ttgttgacttcagaacagaggatggcacagcaaatgctgggtctgattatgaatttactgaaggaactgtggtgtttaagcctggtgatacccagaaggaaatcagagtgggtatcatag
atgatgatatctttgaggaggatgaaaatttccttgtgcatctcagcaatgtcaaagtatcttctgaagcttcagaagatggcatactggaagccaatcatgtttctacacttgcttgcctcg
gatctccctccactgccactgtaactatttttgatgatgaccacgcaggcatttttacttttgaggaacctgtgactcatgtgagtgagagcattggcatcatggaggtgaaagtattgagaa
catctggagctcgaggaaatgttatcgttccatataaaaccatcgaagggactgccagaggtggaggggaggattttgaggacacttgtggagagctcgaattccagaatgatgaaatt
gt
gtgagtggcccgctacctcttctggtggccgcctccctccttcctggcctcccggagctgcgccctttctcactggttctctcttctgccgttttccgtaggatcc

c

hucircSlc8A1His

gtcgacacacttagccgtgttctttgcactttctgcatgtcccccgtctggcctggctgtccccagtggcttccccagtgtgacatggtgcatctctgccttacag
gttgtgacagttggaagtgtcatgtacaacatgcggcgattaagtctttcacccaccttttcaatgggatttcatctgttagttactgtgagtctcttattttcccatgtggaccatgta
caccatcaccatcaccatcac
attgctgagacagaaatggaaggagaaggaaatgaaactggtgaatgtactggatcatattactgtaagaaaggggtgattttgcccatttgggaaccccaagacccttcttttggggac
aaaattgctagagctactgtgtattttgtggccatggtctacatgtttcttggagtctctatcatagctgatcggttcatgtcctctatagaagtcatcacatctcaagaaaaagaaataacca
taaagaaacccaatggagagaccaccaagacaactgtgaggatctggaatgaaacagtttctaacctgaccttgatggccctgggatcttctgctcctgagattctcctttcagtaattga
agtgtgtggccataacttcactgcaggagacctcggtcctagcaccatcgtgggaagtgctgcattcaatatgttcatcattattgcactctgtgtttatgtggtgcctgacggagagacaa
ggaagattaagcatttgcgtgtcttctttgtgacagcagcctggagcatctttgcctacacctggctttacattattttgtctgtcatatctcctggtgttgtggaggtctgggaaggtttgctta
ctttcttcttctttcccatctgtgttgtgttcgcttgggtagcggataggagacttctgttttacaagtatgtctacaagaggtatcgagctggcaagcagagggggatgattattgaacatga
aggagacaggccatcttctaagactgaaattgaaatggacgggaaagtggtcaattctcatgttgaaaatttcttagatggtgctctggttctggaggtggatgagagggaccaagatga
tgaagaagctaggcgagaaatggctaggattctgaaggaacttaagcagaagcatccagataaagaaatagagcaattaatagaattagctaactaccaagtcctaagtcagcagcaa
aaaagtagagcattttatcgcattcaagctactcgcctcatgactggagctggcaacattttaaagaggcatgcagctgaccaagcaaggaaggctgtcagcatgcacgaggtcaacact
gaagtgactgaaaatgaccctgttagtaagatcttctttgaacaagggacatatcagtgtctggagaactgtggtactgtggcccttaccattatccgcagaggtggtgatttgactaacac
tgtgtttgttgacttcagaacagaggatggcacagcaaatgctgggtctgattatgaatttactgaaggaactgtggtgtttaagcctggtgatacccagaaggaaatcagagtgggtatc
atagatgatgatatctttgaggaggatgaaaatttccttgtgcatctcagcaatgtcaaagtatcttctgaagcttcagaagatggcatactggaagccaatcatgtttctacacttgcttgc
ctcggatctccctccactgccactgtaactatttttgatgatgaccacgcaggcatttttacttttgaggaacctgtgactcatgtgagtgagagcattggcatcatggaggtgaaagtattg
agaacatctggagctcgaggaaatgttatcgttccatataaaaccatcgaagggactgccagaggtggaggggaggattttgaggacacttgtggagagctcgaattccagaatgatga
aattgt
gtgagtggcccgctacctcttctggtggccgcctccctccttcctggcctcccggagctgcgccctttctcactggttctctcttctgccgttttccgtaggatcc

d

vector-random-sequence

Ctcgagacacttagccgtgttctttgcactttctgcatgtcccccgtctggcctggctgtccccagtggcttccccagtgtgacatggtgcatctctgccttacag
ggacaattacgtacctttcgtacgtccacgtacgtacaaagtacgtacgtaacgtcgtacgtacgtactttgggttacgtacgtttacgtacttgtacgtaccccgtacggggtacggg
tacgtacgtacgtaccttcgtacgtacccgtacgtacgtacgggtacgtacgtaggtttcgtacgtacgtagggcgtacgtacgtacggtatttcgtacgtacgtaagggaacgtacc
gtacaagtttcag
gtgagtggcccgctacctcttctggtggccgcctccctccttcctggcctcccggagctgcgccctttctcactggttctctcttctgccgttttccgtaggatcc(BamHI)

Supplementary Fig S1. Structure and sequences of circSlc8A1. A. Structure of the constructs. B. Sequence of hucircSlc8a1. C. Sequence of hucircSlc8a1His. D, Sequence of vector.

a

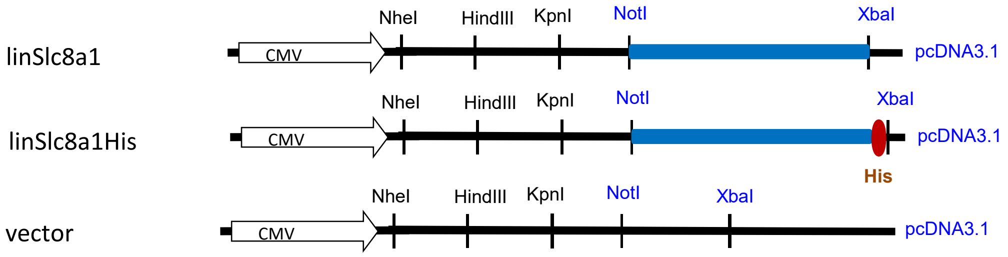

b

Slc8a1-pcDNA3.1 (linSlc8a1)

gccgccacc**atg**tacaacatgcggcgattaagtctttcacccaccttttcaatgggatttcatctgttagttactgtgagtctcttattttcccatgtggaccatgtaattgctgagacagaaat
ggaaggagaaggaaatgaaactggtgaatgtactggatcatattactgtaagaaaggggtgattttgcccatttgggaaccccaagacccttcttttggggacaaaattgctagagctact
gtgtattttgtggccatggtctacatgtttcttggagtctctatcatagctgatcggttcatgtcctctatagaagtcatcacatctcaagaaaaagaaataaccataaagaaacccaatgga
gagaccaccaagacaactgtgaggatctggaatgaaacagtttctaacctgaccttgatggccctgggatcttctgctcctgagattctcctttcagtaattgaagtgtgtggccataacttc
actgcaggagacctcggtcctagcaccatcgtgggaagtgctgcattcaatatgttcatcattattgcactctgtgtttatgtggtgcctgacggagagacaaggaagattaagcatttgcgt
gtcttctttgtgacagcagcctggagcatctttgcctacacctggctttacattattttgtctgtcatatctcctggtgttgtggaggtctgggaaggtttgcttactttcttcttctttcccatctgt
gttgtgttcgcttgggtagcggataggagacttctgttttacaagtatgtctacaagaggtatcgagctggcaagcagagggggatgattattgaacatgaaggagacaggccatcttcta
agactgaaattgaaatggacgggaaagtggtcaattctcatgttgaaaatttcttagatggtgctctggttctggaggtggatgagagggaccaagatgatgaagaagctaggcgagaa
atggctaggattctgaaggaacttaagcagaagcatccagataaagaaatagagcaattaatagaattagctaactaccaagtcctaagtcagcagcaaaaaagtagagcattttatcg
cattcaagctactcgcctcatgactggagctggcaacattttaaagaggcatgcagctgaccaagcaaggaaggctgtcagcatgcacgaggtcaacactgaagtgactgaaaatgacc
ctgttagtaagatcttctttgaacaagggacatatcagtgtctggagaactgtggtactgtggcccttaccattatccgcagaggtggtgatttgactaacactgtgtttgttgacttcagaac
agaggatggcacagcaaatgctgggtctgattatgaatttactgaaggaactgtggtgtttaagcctggtgatacccagaaggaaatcagagtgggtatcatagatgatgatatctttgag
gaggatgaaaatttccttgtgcatctcagcaatgtcaaagtatcttctgaagcttcagaagatggcatactggaagccaatcatgtttctacacttgcttgcctcggatctccctccactgcca
ctgtaactatttttgatgatgaccacgcaggcatttttacttttgaggaacctgtgactcatgtgagtgagagcattggcatcatggaggtgaaagtattgagaacatctggagctcgagga
aatgttatcgttccatataaaaccatcgaagggactgccagaggtggaggggaggattttgaggacacttgtggagagctcgaattccagaatgatgaaattgtgttg**tga**tctaga

c

Slc8a1-His-pcDNA3.1 (linSlc8a1His)

gccgccaccatgtacaacatgcggcgattaagtctttcacccaccttttcaatgggatttcatctgttagttactgtgagtctcttattttcccatgtggaccatgtaattgctgagacagaaat
ggaaggagaaggaaatgaaactggtgaatgtactggatcatattactgtaagaaaggggtgattttgcccatttgggaaccccaagacccttcttttggggacaaaattgctagagctact
gtgtattttgtggccatggtctacatgtttcttggagtctctatcatagctgatcggttcatgtcctctatagaagtcatcacatctcaagaaaaagaaataaccataaagaaacccaatgga
gagaccaccaagacaactgtgaggatctggaatgaaacagtttctaacctgaccttgatggccctgggatcttctgctcctgagattctcctttcagtaattgaagtgtgtggccataacttc
actgcaggagacctcggtcctagcaccatcgtgggaagtgctgcattcaatatgttcatcattattgcactctgtgtttatgtggtgcctgacggagagacaaggaagattaagcatttgcg
tgtcttctttgtgacagcagcctggagcatctttgcctacacctggctttacattattttgtctgtcatatctcctggtgttgtggaggtctgggaaggtttgcttactttcttcttctttcccatctg
tgttgtgttcgcttgggtagcggataggagacttctgttttacaagtatgtctacaagaggtatcgagctggcaagcagagggggatgattattgaacatgaaggagacaggccatcttct
aagactgaaattgaaatggacgggaaagtggtcaattctcatgttgaaaatttcttagatggtgctctggttctggaggtggatgagagggaccaagatgatgaagaagctaggcgaga
aatggctaggattctgaaggaacttaagcagaagcatccagataaagaaatagagcaattaatagaattagctaactaccaagtcctaagtcagcagcaaaaaagtagagcattttatc
gcattcaagctactcgcctcatgactggagctggcaacattttaaagaggcatgcagctgaccaagcaaggaaggctgtcagcatgcacgaggtcaacactgaagtgactgaaaatgac
cctgttagtaagatcttctttgaacaagggacatatcagtgtctggagaactgtggtactgtggcccttaccattatccgcagaggtggtgatttgactaacactgtgtttgttgacttcagaa
cagaggatggcacagcaaatgctgggtctgattatgaatttactgaaggaactgtggtgtttaagcctggtgatacccagaaggaaatcagagtgggtatcatagatgatgatatctttga
ggaggatgaaaatttccttgtgcatctcagcaatgtcaaagtatcttctgaagcttcagaagatggcatactggaagccaatcatgtttctacacttgcttgcctcggatctccctccactgcc
actgtaactatttttgatgatgaccacgcaggcatttttacttttgaggaacctgtgactcatgtgagtgagagcattggcatcatggaggtgaaagtattgagaacatctggagctcgagg
aaatgttatcgttccatataaaaccatcgaagggactgccagaggtggaggggaggattttgaggacacttgtggagagctcgaattccagaatgatgaaattgtgttg**caccatcacca**
tcaccatcactgatctaga

Supplementary Fig S2. Structure and sequences of linear Slc8A1. A. Structure of the constructs. B. Sequence of linSlc8a1. C. Sequence of linSlc8a1His.

a

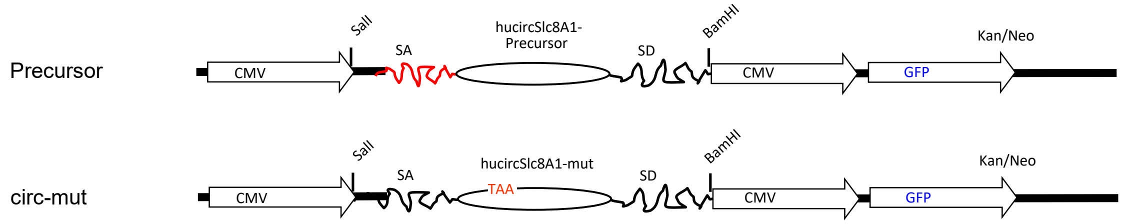

SA, splice acceptor; SD, splice donor

b

<u>hucircSlc8A1-Precursor (Precursor)</u>

gtcgac acacttagccgtgttctttgcactttctgcatgtcccccgtctggcctggctgtccccagtggcttccccagtgtgacat**<u>aaatttaattaaaaaatttt</u>**
gttgtgacagttggaagtgtcatgtacaacatgcggcgattaagtctttcacccaccttttcaatgggatttcatctgttagttactgtgagtctcttattttcccatgtggaccatgtaat
tgctgagacagaaatggaaggagaaggaaatgaaactggtgaatgtactggatcatattactgtaagaaaggggtgattttgcccatttgggaaccccaagacccttcttttgggg
acaaaattgctagagctactgtgtattttgtggccatggtctacatgtttcttggagtctctatcatagctgatcggttcatgtcctctatagaagtcatcacatctcaagaaaaagaaa
taaccataaagaaacccaatggagagaccaccaagacaactgtgaggatctggaatgaaacagtttctaacctgaccttgatggccctgggatcttctgctcctgagattctcctttc
agtaattgaagtgtgtggccataacttcactgcaggagacctcggtcctagcaccatcgtgggaagtgctgcattcaatatgttcatcattattgcactctgtgtttatgtggtgcctga
cggagagacaaggaagattaagcatttgcgtgtcttctttgtgacagcagcctggagcatctttgcctacacctggctttacattattttgtctgtcatatctcctggtgttgtggaggtc
tgggaaggtttgcttactttcttcttctttcccatctgtgttgtgttcgcttgggtagcggataggagacttctgttttacaagtatgtctacaagaggtatcgagctggcaagcagagg
gggatgattattgaacatgaaggagacaggccatcttctaagactgaaattgaaatggacgggaaagtggtcaattctcatgttgaaaatttcttagatggtgctctggttctggag
gtggatgagagggaccaagatgatgaagaagctaggcgagaaatggctaggattctgaaggaacttaagcagaagcatccagataaagaaatagagcaattaatagaattagc
taactaccaagtcctaagtcagcagcaaaaaagtagagcattttatcgcattcaagctactcgcctcatgactggagctggcaacattttaaagaggcatgcagctgaccaagcaa
ggaaggctgtcagcatgcacgaggtcaacactgaagtgactgaaaatgaccctgttagtaagatcttctttgaacaagggacatatcagtgtctggagaactgtggtactgtggcc
cttaccattatccgcagaggtggtgatttgactaacactgtgtttgttgacttcagaacagaggatggcacagcaaatgctgggtctgattatgaatttactgaaggaactgtggtgtt
taagcctggtgatacccagaaggaaatcagagtgggtatcatagatgatgatatctttgaggaggatgaaaatttccttgtgcatctcagcaatgtcaaagtatcttctgaagcttca
gaagatggcatactggaagccaatcatgtttctacacttgcttgcctcggatctccctccactgccactgtaactatttttgatgatgaccacgcaggcatttttacttttgaggaacct
gtgactcatgtgagtgagagcattggcatcatggaggtgaaagtattgagaacatctggagctcgaggaaatgttatcgttccatataaaccatcgaagggactgccagaggtgg
aggggaggattttgaggacacttgtggagagctcgaattccagaatgatgaaattgt
gtgagtggcccgctacctcttctggtggccgcctccctccttcctggcctcccggagctgcgccctttctcactggttctctcttctgccgttttccgtaggatcc

c

<u>hucircSlc8A1-mut (circ-mut)</u>

gtcgacacacttagccgtgttctttgcactttctgcatgtcccccgtctggcctggctgtccccagtggcttccccagtgtgacatggtgcatctctgccttacag
gttgtgacagttggaagtgtcatgtacaacatgcggcgattaagtctttcacccaccttttcaatgggatttcatctgttagttactgtgagtctcttattttcccatgtggaccatgtaat
tgctgagacagaaatggaaggagaaggaaatgaaactggtgaatgtactggatcatattactgtaagaaaggggtgattttgcccatttgggaaccccaagacccttcttttgggg
acaaaattgctagagctactgtgtattttgtggccatggtctacatgtttcttggagtctctatcatagctgatcggttc<u>TAA</u>tcctctatagaagtcatcacatctcaagaaaaagaa
ataaccataaagaaacccaatggagagaccaccaagacaactgtgaggatctggaatgaaacagtttctaacctgaccttgatggccctgggatcttctgctcctgagattctcctt
tcagtaattgaagtgtgtggccataacttcactgcaggagacctcggtcctagcaccatcgtgggaagtgctgcattcaatatgttcatcattattgcactctgtgtttatgtggtgcct
gacggagagacaaggaagattaagcatttgcgtgtcttctttgtgacagcagcctggagcatctttgcctacacctggctttacattattttgtctgtcatatctcctggtgttgtggag
gtctgggaaggtttgcttactttcttcttctttcccatctgtgttgtgttcgcttgggtagcggataggagacttctgttttacaagtatgtctacaagaggtatcgagctggcaagcaga
gggggatgattattgaacatgaaggagacaggccatcttctaagactgaaattgaaatggacgggaaagtggtcaattctcatgttgaaaatttcttagatggtgctctggttctgg
aggtggatgagagggaccaagatgatgaagaagctaggcgagaaatggctaggattctgaaggaacttaagcagaagcatccagataaagaaatagagcaattaatagaatta
gctaactaccaagtcctaagtcagcagcaaaaaagtagagcattttatcgcattcaagctactcgcctcatgactggagctggcaacattttaaagaggcatgcagctgaccaagc
aaggaaggctgtcagcatgcacgaggtcaacactgaagtgactgaaaatgaccctgttagtaagatcttctttgaacaagggacatatcagtgtctggagaactgtggtactgtgg
cccttaccattatccgcagaggtggtgatttgactaacactgtgtttgttgacttcagaacagaggatggcacagcaaatgctgggtctgattatgaatttactgaaggaactgtggt
gtttaagcctggtgatacccagaaggaaatcagagtgggtatcatagatgatgatatctttgaggaggatgaaaatttccttgtgcatctcagcaatgtcaaagtatcttctgaagct
tcagaagatggcatactggaagccaatcatgtttctacacttgcttgcctcggatctccctccactgccactgtaactatttttgatgatgaccacgcaggcatttttacttttgaggaa
cctgtgactcatgtgagtgagagcattggcatcatggaggtgaaagtattgagaacatctggagctcgaggaaatgttatcgttccatataaaccatcgaagggactgccagagg
tggaggggaggattttgaggacacttgtggagagctcgaattccagaatgatgaaattgt
gtgagtggcccgctacctcttctggtggccgcctccctccttcctggcctcccggagctgcgccctttctcactggttctctcttctgccgttttccgtaggatcc

Supplementary Fig S3. Structure and sequences of mutated circSlc8A1. A. Structure of the constructs. B. Sequence of Precursor. C. Sequence of circ-mut.

a

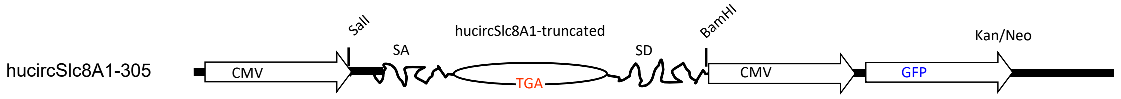

SA, splice acceptor; SD, splice donor

b

CP159.hucircSlc8A1-305
gtcgacacacttagccgtgttctttgcactttctgcatgtcccccgtctggcctggctgtccccagtggcttccccagtgtgacatggtgcatctctgccttacag
gttgtgacagttggaagtgtcatgtacaacatgcggcgattaagtctttcacccaccttttcaatgggatttcatctgttagttactgtgagtctcttattttcccatgtggaccatgtaattgctgagacagaaatg
gaaggagaaggaaatgaaactggtgaatgtactggatcatattactgtaagaaaggggtgattttgcccatttgggaaccccaagacccttcttttggggacaaaattgctagagctactgtgtattttgtgg
ccatggtctacatgtttcttggagtctctatcatagctgatcggttcatgtcctctatagaagtcatcacatctcaagaaaaagaaataaccataaagaaacccaatggagagaccaccaagacaactgtg
aggatctggaatgaaacagtttctaacctgaccttgatggccctgggatcttctgctcctgagattctcctttcagtaattgaagtgtgtggccataacttcactgcaggagacctcggtcctagcaccatcgtg
ggaagtgctgcattcaatatgttcatcattattgcactctgtgtttatgtggtgcctgacggagagacaaggaagattaagcatttgcgtgtcttctttgtgacagcagcctggagcatctttgcctacacctggct
ttacattattttgtctgtcatatctcctggtgttgtggaggtctgggaaggtttgcttactttcttcttctttcccatctgtgttgtgttcgcttgggtagcggataggagacttctgttttacaagtatgtctacaagaggta
tcgagctggcaagcagaggggatgattattgaacatgaaggagacaggccatcttctaagactgaaattgaaatggacgggaaagtggtcaattctcatgttgaaaatttcttagat
TGAgagggaccaagatgatgaagaagctaggcgagaaatggctaggattctgaaggaacttaagcagaagcatccagataaagaaatagagcaattaatagaattagctaactaccaagtccta
agtcagcagcaaaaaagtagagcattttatcgcattcaagctactcgcctcatgactggagctggcaacattttaaagaggcatgcagctgaccaagcaaggaaggctgtcagcatgcacgaggtcaa
cactgaagtgactgaaaatgaccctgttagtaagatcttctttgaacaagggacatatcagtgtctggagaactgtggtactgtggcccttaccattatccgcagaggtggtgatttgactaacactgtgtttgt
tgacttcagaacagaggatggcacagcaaatgctgggtctgattataaggacgacgatgacaaggaatttactgaaggaactgtggtgtttaagcctggtgatacccagaaggaaatcagagtgggta
tcatagatgatgatatctttgaggaggatgaaaatttccttgtgcatctcagcaatgtcaaagtatcttctgaagcttcagaagatggcatactggaagccaatcatgtttctacacttgcttgcctcggatctcc
ctccactgccactgtaactatttttgatgatgaccacgcaggcatttttacttttgaggaacctgtgactcatgtgagtgagagcattggcatcatggaggtgaaagtattgagaacatctggagctcgagga
aatgttatcgttccatataaaaccatcgaagggactgccagaggtggaggggaggattttgaggacacttgtggagagctcgaattccagaatgatgaaattgt
gtgagtggcccgctacctcttctggtggccgcctccctccttcctggcctcccggagctgcgccctttctcactggttctctcttctgccgttttccgtaggatcc

Supplementary Fig S4. Structure and sequences of mutated circSlc8A1. A. Structure of the mutation construct. B. Sequence of circSlc8A1 truncation/mutation deleting the C-terminal fragment.

a **Primer sequences**

32.74.HuMu.cir.Slc8a1-F 5’ tgtggagagctcgaattccagaa
32.75.HuMu.cir.Slc8a1-R 5’ atcacccctttcttacagtaatat (239 bp)
32.76.HuMu.cir.Slc8a1-0944F 5’ attccagaatgatgaaattgttag (combine with 32.75.HuMu.cir.Slc8a1-R)
32.77.HuMu.cir.Slc8a1-5232F 5’ attccagaatgatgaaattgtgt (can combine with 32.75.HuMu.cir.Slc8a1-R)
34.15.huMu.cir.lin.Slc8a1-F 5’ cttttggggacaaaattgctagag
34.16.huMu.cir.lin.Slc8a1-R 5’ aatctcaggagcagaagatcccag (239 bp)
34.17.huMu.linear.Slc8a1-F 5’ gatgaggagtatgagaaaaacaag
34.18.huMu.linear.Slc8a1-R. 5’ tctttgctggtcagtggctgcttg (239 bp)
34.56.hu-circSlc8a1-probe-F 5’ **gtcattacgg**ttccagaa
34.57.hu-circSlc8a1-probe-R 5’ gggcccgggaaaacactt (large scale, 180 bp)
36.18.Genomtyping1-F 5’ gcactttctgcatgtcccccgtc
36.23.hucircSlc8A1-genomtyping-R. 5’ cgcatgttgtacatgacacttcc (+ 36.18.G.., 116 bp)
37.1.Hu-cirSlc8A1-F 5’ tgccagaggtggaggggaggat
37.2.Hu-cirSlc8A1-R 5’ atcgccgcatgttgtacatgac (109 bp)

42.26.hu-MT-ATP6-F, 5’ CTGTTCGCTTCATTCATTGCC
42.27.hu-MT-ATP6-R, 5’ GATGAGATATTTGGAGGTGGG
42.28.HU-MT-ATP8-F, 5’ CCAACTAAATACTACCGTATGG
42.29.HU-MT-ATP8-R, 5’ GGAGGTAGGTGGTAGTTTGTG
42.30.HU-MT-ND1-F, 5’ CCAACCTCCTACTCCTCATTG
42.31.HU-MT-ND1-R, 5’ GTTGGGGCCTTTGCGTAGTTG
42.32.HU-MT-ND2-F, 5’ CTCTGACATCCGGCCTGCTTC
42.33.HU-MT-ND2-R, 5’ GAGAGTGAGGAGAAGGCTTAC
42.34.HU-MT-ND3-F, 5’ TCAACACCCTCCTAGCCTTAC
42.35.HU-MT-ND3-R, 5’ GTCGAAGCCGCACTCGTAAGG
42.36.HU-MT-ND4-F, 5’ CTCCTACCCCTCACAATCATG
42.37.HU-MT-ND4-R, 5’ GGAGATTTGTAGGGAGATTAG
42.38.HU-MT-ND4L-F, 5’ CATTTACCATCTCACTTCTAG
42.39.HU-MT-ND4L-R, 5’ GAGTAGCTATAATGAACAGCG
42.40.HU-MT-ND5-F, 5’ CCACAACCCAAACAACCCAGC
42.41.HU-MT-ND5-R, 5’ GTGAGAATTCTATGATGGACC
42.42.HU-MT-ND6-F, 5’ TGTTAGCGGTGTGGTCGGGTG
42.43.HU-MT-ND6-R, 5’ CCAAAGACAACCATCATTCCC
42.44.HU-MT-CO1-F, 5’ GCCGAGCTGGGCCAGCCAGGC
42.45.HU-MT-CO1-R, 5’ GATGGGTATTACTATGAAGAAG
42.46.HU-MT-CO2-F, 5’ CTACTTCCCCTATCATAGAAG
42.47.HU-MT-CO2-R, 5’ AGTGTTAGGAAAAGGGCATAC
42.48.HU-MT-CO3-F, 5’ CAGCCCATGACCCCTAACAGG
42.49.HU-MT-CO3-R, 5’ GTAGGCCTAGTATGAGGAGCG
42.50.HU-MT-CYB-F, 5’ CTTGGCGCCTGCCTGATCCTC
42.51.HU-MT-CYB-R, 5’ CTCGAGTGATGTGGGCGATTG

b **siRNA sequences**

1. si-Hu-circSlc8A1-1
5’ *gaaauuguguugugacaguuu*
3’ cccuuuaacacaacacuguca

2. si-Hu-circSlc8A1-2
5’ gau*gaaauuguguugugacuu*
3’ cccuacuuuaacacaacacug

3. si-Hu-lincirSlc8A1-1 (si-both-1)
5’ ggaaugaaacaguuucuaa*uu*
3’ ggccuuacuuugucaaagauu

4. si-Hu-lincirSlc8A1-2 (si-both-2)
5’ ggacgggaaaguggucaau*uu*
3’ ggccugcccuuucaccaguua

5. si-Hu-linSlc8A1-1
5’ gccaaccugucuucaggaa*uu*
3’ ggcgguuggacagaaguccuu

6. si-Hu-linSlc8A1-2
5’ ggagaggcgcauugcagaa*uu*
3’ ggccucuccgcguaacgucuu

Supplementary Fig S5. Sequences of primers and siRNA. A. Sequences of primers used in the study. B. Sequence of siRNA used in the study.

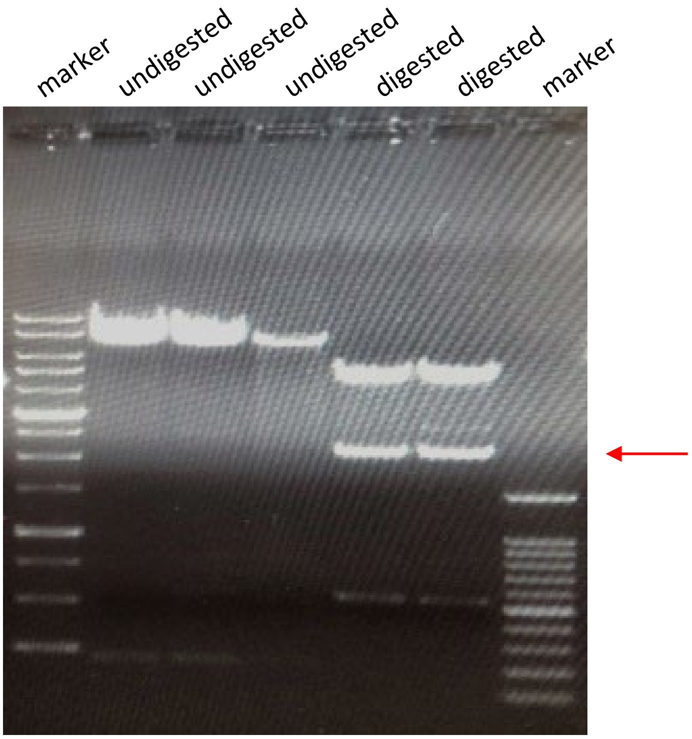

Figure S6. Isolation of DNA for generation of transgenic mice. circSLC8A1-containing DNA fragment (arrow) was visualized on DNA gel and purified for microinjection.

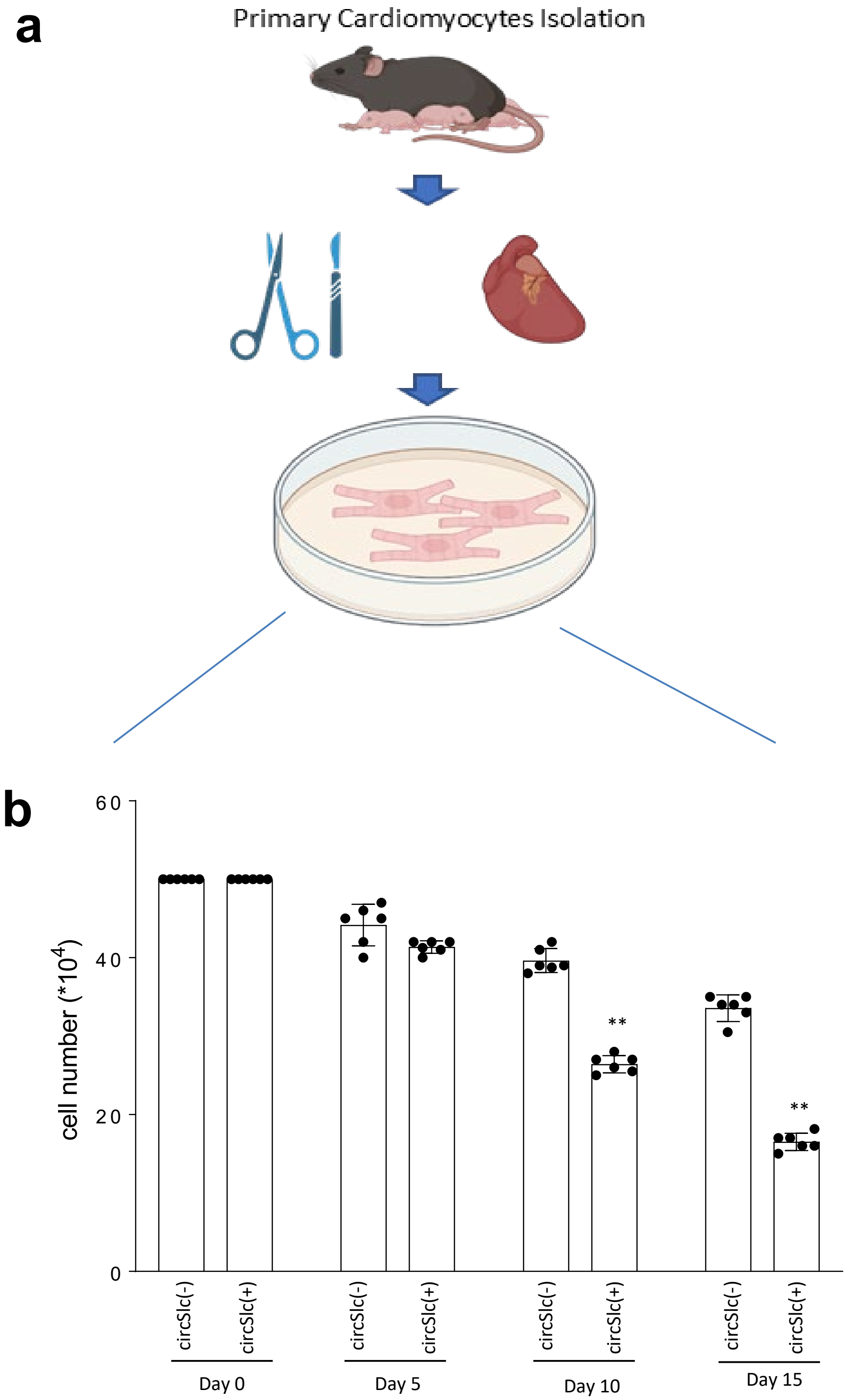

Figure S7. Effect of circSlc8a1 on cell viability. (a) Primary cardiac myocytes were isolated from transgenic mice. **(b)** Cells were cultured at serum free condition for 15 days. Cells were counted every 5 days for survival assay. CircSLC8A1 decreased primary cardiac myocyte survival. n = 6, **$p<0.01$ vs. negative control ± SD.

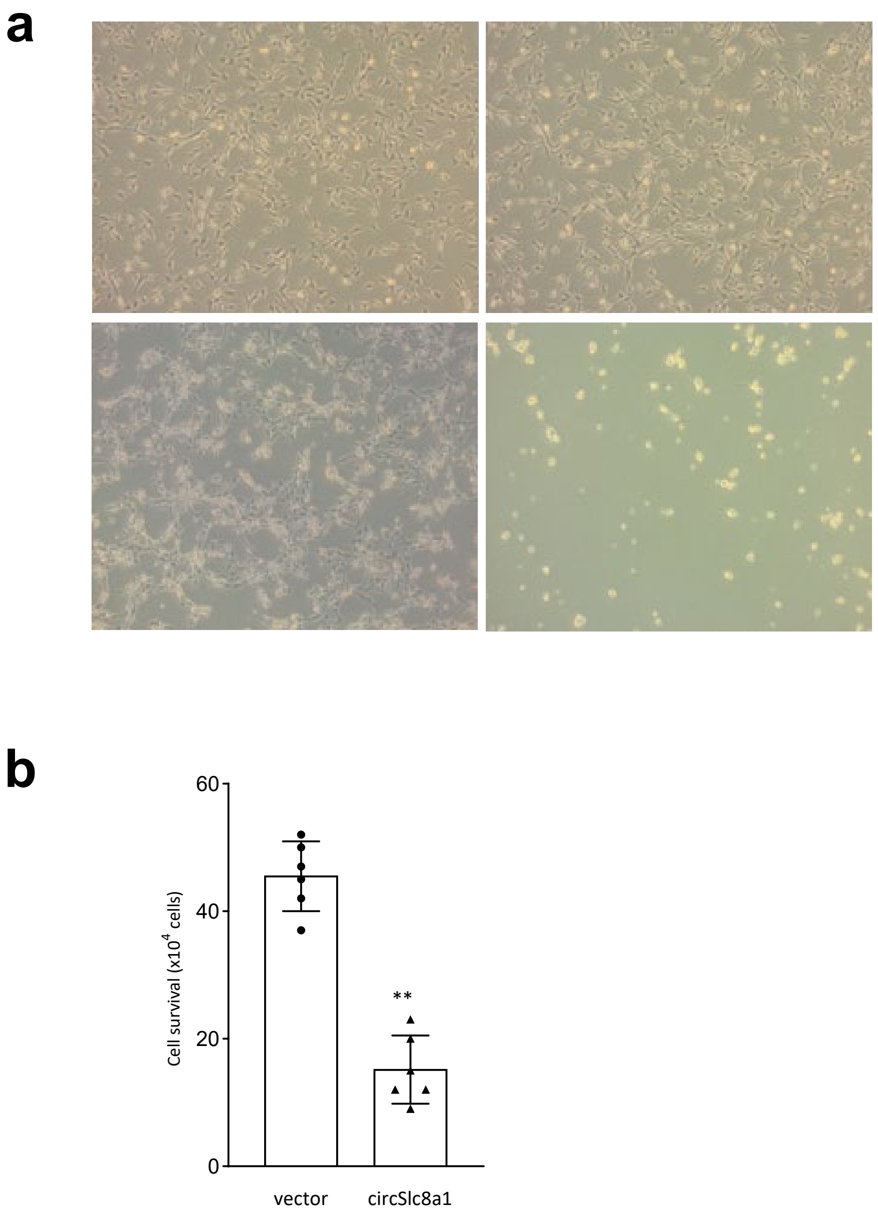

Figure S8. CircSLC8A1 decreased survival of HL-1 cardiomyocytes cell line.
(a) Representative photos showing decreased survival in circSLC8A1 overexpression group on Day 15 of serum free culture. **(b)** Statistical analysis of survival assay. CircSLC8A1 significantly decreased survival compared to vector control. n = 6, **$p<0.01$ vs. vector control ± SD.

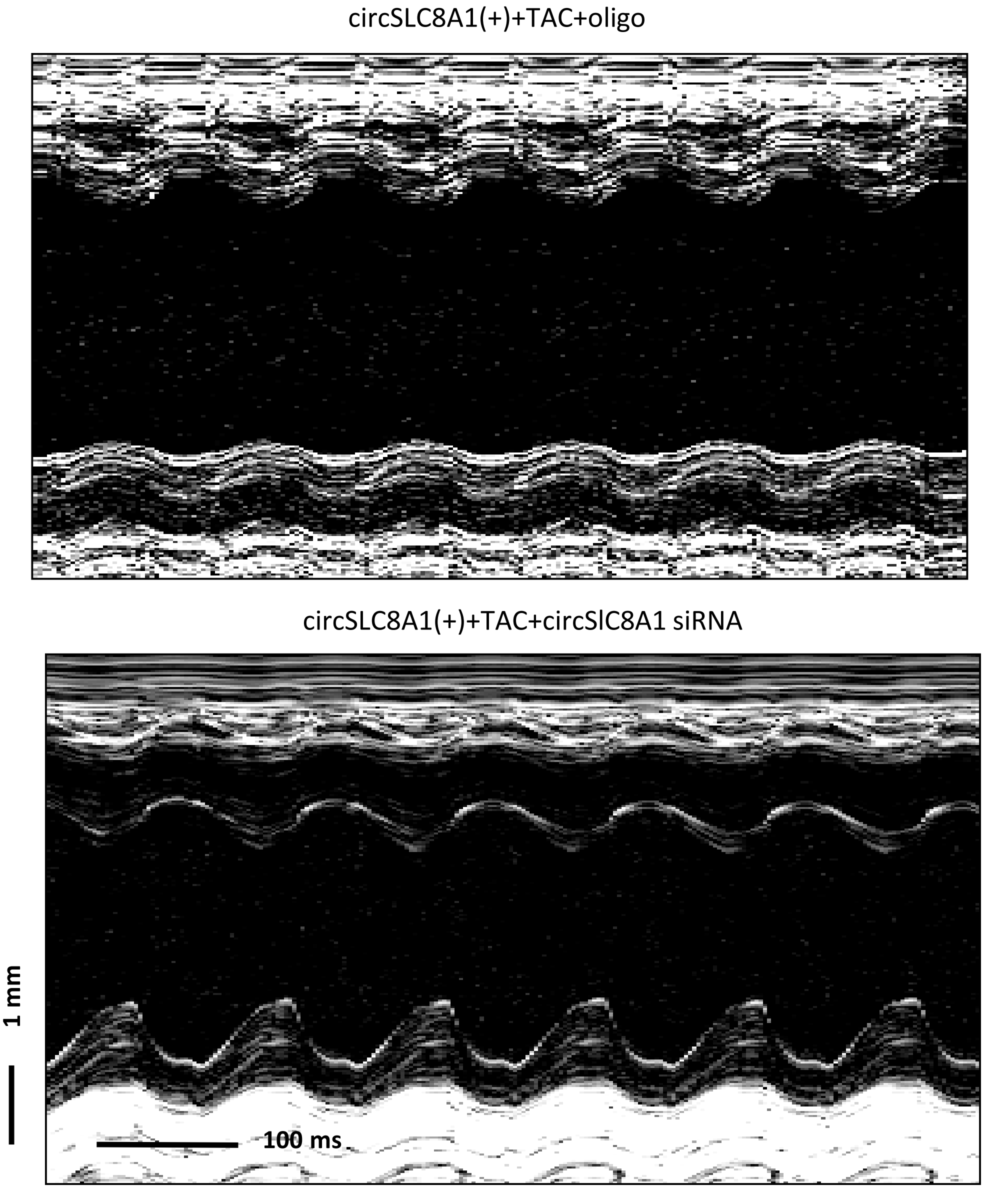

Figure S9. Representative M-mode echocardiography images from the transgenic mice who underwent TAC with or without siRNA injection. CircSLC8a1 siRNA delivery improved heart functions.

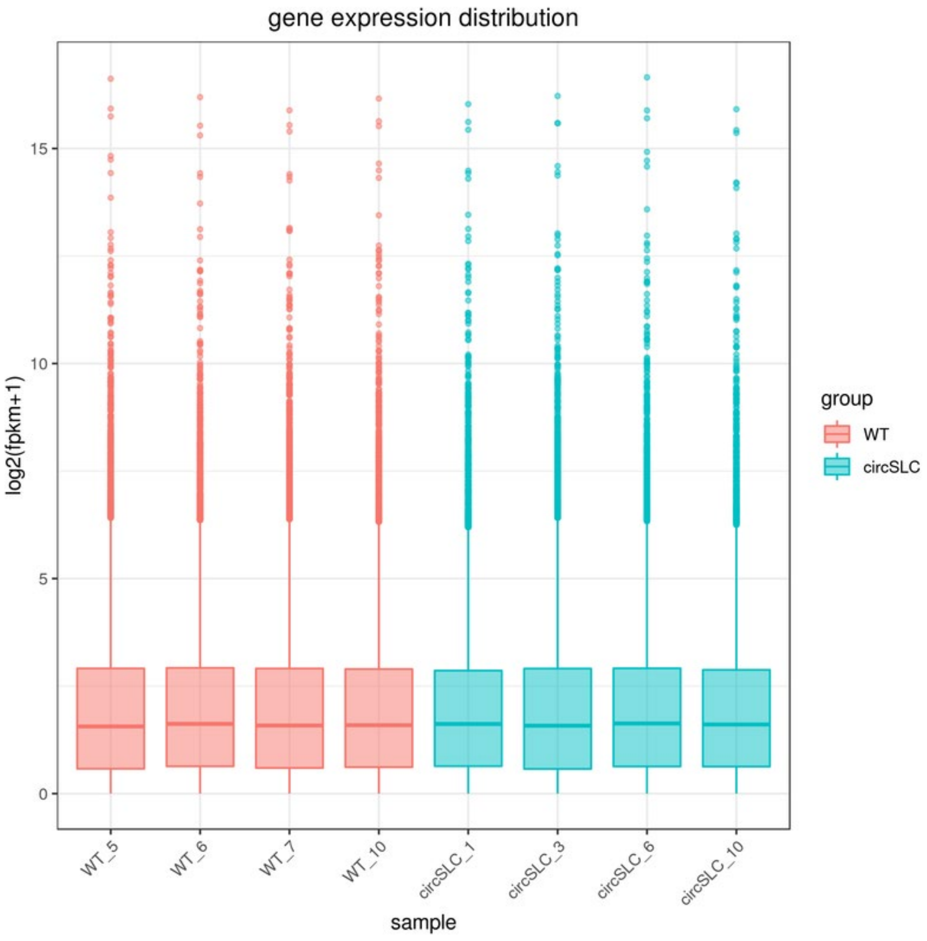

Figure S10. Distribution of gene expression (comparing difference of samples).

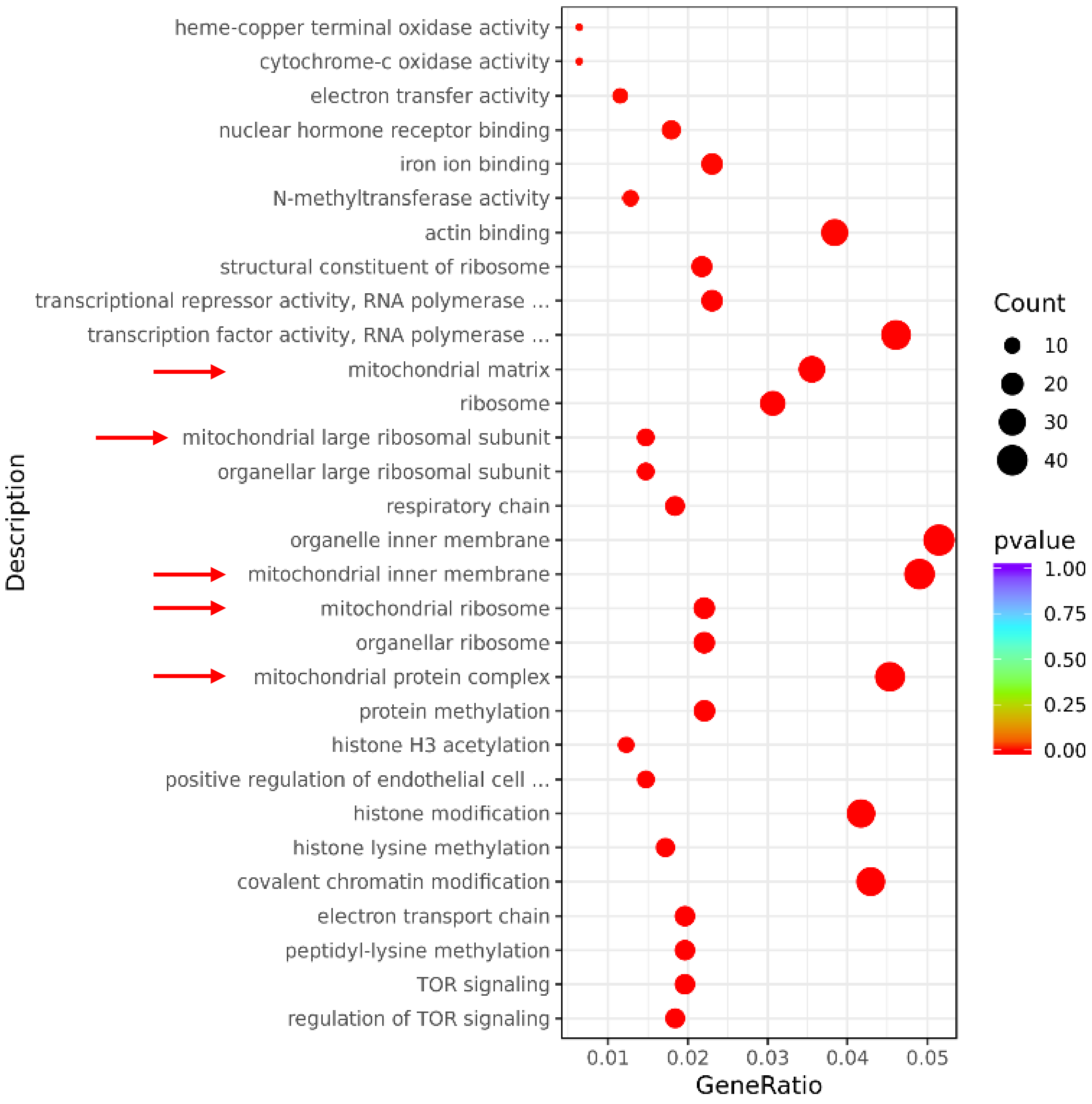

Figure S11. Enrichment of pathways. It shows mitochondrial associated genes are involved in the enriched pathways (red arrows).

a

Mus-circSlc8A1

gtcgacacacttagccgtgttctttgcactttctgcatgtcccccgtctggcctggctgtccccagtggcttccccagtgtgacatggtgcatctctgccttacaggttggagcagttggaagtct
tattgtacaac**atg**cttcgattaagtctcccacccaatgtttcaatgggatttcgtctggtagctctggtggctctcttgttttcccatgttgaccatataactgcagatacagaggcagaaaca
ggaggaaatgaaaccactgaatgtactggctcatattactgtaagaaaggggtgatcttgcccatttgggaaccccaagacccatcttttggggacaaaattgctagagcaactgtgtattt
tgtggccatggtctacatgttccttggagtttctattattgcagaccggtttatgtcctctatagaggtcatcacctctcaagagaaagaaataacgataaagaaaccgaatggagagaccac
caagacgacggtgagaatctggaacgagactgtgtcgaacctgaccttgatggccctgggatcttctgctcctgagattctcctgtcagtcattgaagtgtgcggccataacttcaccgcagg
ggacctgggtcccagcaccatcgtgggaagtgctgcctttaacatgttcatcataatcgcactctgtgtttacgtggtccctgatggagagacaaggaagatcaagcatctgcgtgtgttcttt
gtgacagcagcctggagcatctttgcctatacctggctttatataatcttgtctgtcagctctcctggagttgtggaggtctgggaaggcttgcttactttcttcttctttcccatctgcgttgtgtt
cgcgtgggtagcagacaggcggcttctcttttacaagtatgtctacaagcggtacagggccggcaagcagaggggggatgatcattgaacatgaaggagacagaccagcttccaaaactga
aatcgaaatggatgggaaagtggtcaactctcatgttgacaatttcttagatggggctctggttttggaagttgatgagagggaccaagatgatgaggaagccaggcgtgagatggcaag
gattctgaaggaacttaagcagaagcatcctgagaaagaaattgagcaattaatagaattagccaactaccaggtcctaagtcaacagcagaaaagccgagcattttacaggattcaagc
tactcgcctgatgaccggagctggcaacatcttgaagaggcacgcagctgatcaagcaaggaaggctgtcagtatgcatgaagtcaacatggaaatggctgaaaacgacccagtcagta
agatcttctttgagcaaggaacataccagtgtctagagaactgtggtactgtggccctcaccattatgcgcagagggggcgacttgagcaccactgtgtttgttgacttcaggacagaagac
ggcacagccaatgctgggtctgattatgaattcacggaagggactgtgatcttcaaaccaggggagacccagaaggaaatcagagttggcatcattgatgatgatatctttgaagaagatg
aaaacttccttgtgcatcttagcaatgtcagagtctcttcagatgtttcagaagatggcatactagaatccaatcacgcttcttcaattgcttgtcttgggtcacccagcactgccaccataacc
atttttgatgatgaccatgcaggcatctttacatttgaggaacccgtgactcacgtgagcgagagcattggcatcatggaggtgaaggttttgagaacctctggagctcgaggaaatgttatc
attccctacaaaactattgaaggcacagcccgaggtggaggggaagactttgaggacacctgtggagagctcgaattccagaacgatgaaatagtgtgagtggcccgctacctcttctggt
ggccgcctccctccttcctggcctcccggagctgcgccctttctcactggttctctcttctgccgttttccgtaggatcc

Mus-circSlc8A1-stop codon

gtcgacacacttagccgtgttctttgcactttctgcatgtcccccgtctggcctggctgtccccagtggcttccccagtgtgacatggtgcatctctgccttacaggttggagcagttggaagtct
tattgtacaac**atg**cttcgattaagtctcccacccaatgtttcaatgggatttcgtctggtagctctggtggctctcttgttttcccatgttgaccatataactgcagatacagaggcagaaaca
ggaggaaatgaaaccactgaatgtactggctcatattactgtaagaaaggggtgatcttgcccatttgggaaccccaagacccatcttttggggacaaaattgctagagcaactgtgtattt
tgtggccatggtctacatgttccttggagtttctattattgcagaccggtttatgtcctctatagaggtcatcacctctcaagagaaagaaataacgataaagaaaccgaatggagagaccac
caagacgacggtgagaatctggaacgagactgtgtcgaacctgaccttgatggccctgggatcttctgctcctgagattctcctgtcagtcattgaagtgtgcggccataacttcaccgcagg
ggacctgggtcccagcaccatcgtgggaagtgctgcctttaacatgttcatcataatcgcactctgtgtttacgtggtccctgatggagagacaaggaagatcaagcatctgcgtgtgttcttt
gtgacagcagcctggagcatctttgcctatacctggctttatataatcttgtctgtcagctctcctggagttgtggaggtctgggaaggcttgcttactttcttcttctttcccatctgcgttgtgtt
cgcgtgggtagcagacaggcggcttctcttttacaagtatgtctacaagcggtacagggccggcaagcagaggggggatgatcattgaacatgaaggagacagaccagcttccaaaactga
aatcgaaatggatgggaaagtggtcaactctcatgttgacaatttcttagatggggctctggttttggaagttgatgagagggaccaagatgatgaggaagccaggcgtgagatggcaag
gattctgaaggaacttaagcagaagcatcctgagaaagaaattgagcaattaatagaattagccaactaccaggtcctaagtcaacagcagaaaagccgagcattttacaggattcaagc
tactcgcctgatgaccggagctggcaacatcttgaagaggcacgcagctgatcaagcaaggaaggctgtcagtatgcatgaagtcaacatggaaatggctgaaaacgacccagtcagta
agatcttctttgagcaaggaacataccagtgtctagagaactgtggtactgtggccctcaccattatgcgcagagggggcgacttgagcaccactgtgtttgttgacttcaggacagaagac
ggcacagccaatgctgggtctgattatgaattcacggaagggactgtgatcttcaaaccaggggagacccagaaggaaatcagagttggcatcattgatgatgatatctttgaagaagatg
aaaacttccttgtgcatcttagcaatgtcagagtctcttcagatgtttcagaagatggcatactagaatccaatcacgcttcttcaattgcttgtcttgggtcacccagcactgccaccataacc
atttttgatgatgaccatgcaggcatctttacatttgaggaacccgtgactcacgtgagcgagagcattggcatcatggaggtgaaggttttgagaacctctggagctcgaggaaatgttatc
attccctacaaaactattgaaggcacagcccgaggtggaggggaagactttgaggacacctgtggagagctcgaattccagaacgatgaa**taa**gtgtgagtggcccgctacctcttctggt
ggccgcctccctccttcctggcctcccggagctgcgccctttctcactggttctctcttctgccgttttccgtaggatcct

b

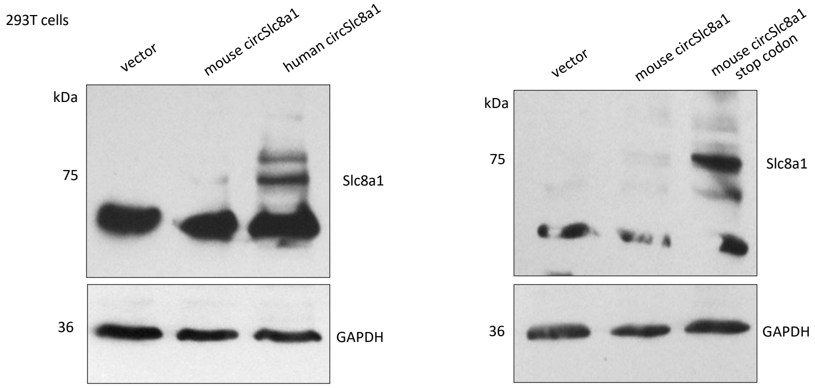

Supplementary Fig S12. Expression of mutated mouse Slc8A1. A. Upper, Sequence of mouse circSlc8a1. Lower, Sequence of mouse circSlc8a1 engineered with a stop codon TAA. B. Left, Western blot detected a protein band at 75 kDa when 293T cells were transfected with human circSlc8a1. At the same location, no protein band was detected when the cells were transfected with mouse circSlc8a1. Right. A protein band was detected when the cells were transfected with mouse circSlc8a1, in which a stop codon was engineered at the end of the circRNA sequence. Potential glycosylation of the proteins resulted in the detection of more than one band.

Supplementary Tables

Table S1. Information of cardiac samples

	sample No	heart function	heart function						
Normal, heart donation	n=20								
		LVEF	LVFS	AGE	SEX		PCR		IHC
brain trauma	61			46	M		real-time PCR		IHC staining
brain hemorrhage	62			37	M		real-time PCR		
car accident	68			47	M		real-time PCR		
brain hemorrhage	69			32	M		real-time PCR		
car accidnet	70			49	M		real-time PCR		
car accident	71			57	M		real-time PCR		
Brain tumour	77			23	M		real-time PCR		
brain hemorrhage	79			42	M		real-time PCR		IHC staining
car accident	94			47	F		real-time PCR		IHC staining
car accident	95			24	M		real-time PCR		IHC staining
brain hemorrhage	104			37	F		real-time PCR		IHC staining
car accident	107			61	M		real-time PCR		IHC staining
trauma	119			19	M		real-time PCR		IHC staining
trauma	132			34	M		real-time PCR		IHC staining
brain deiseases	137			35	M		real-time PCR		IHC staining
brain hemorrhage	139			23	M		real-time PCR		IHC staining
brain hemorrhage	145			50	M		real-time PCR		
brain hemorrhage	146			40	M		real-time PCR		
brain hemorrhage	147			55	M		real-time PCR		
brain hemorrhage	153			22	F		real-time PCR		
HF	n=20						PCR		staining
CAD	HF9	36		66	M		real-time PCR		IHC staining
DCM	14L	29		37	M		real-time PCR		IHC staining
DCM	15L	32		52	M		real-time PCR		
DCM	16HF	44		41	M		real-time PCR		IHC staining
MS	17 HF	24		29	F		real-time PCR		
DCM	18HF	23		51	F		real-time PCR		
DCM	19HF	16.9		45	M		real-time PCR		
DCM	HF25	28	14	40	M		real-time PCR		IHC staining
DCM	HF31	24		31	M		real-time PCR		IHC staining
CAD	HF32	21		68	M		real-time PCR		IHC staining
DCM	HF33	33		28	M		real-time PCR		IHC staining
DCM	HF34	19		38	F		real-time PCR		
DCM	HF35	27		53	M		real-time PCR		
DCM	HF2	27		23	M		real-time PCR		IHC staining
DCM	HF3	21		55	M		real-time PCR		IHC staining
CAD	HF4	27		44	M		real-time PCR		IHC staining
DCM	HF5	22		42	M		real-time PCR		IHC staining
MS	HF6	27		54	M		real-time PCR		IHC staining
DCM	HF7	28		55	M		real-time PCR		IHC staining
DCM	HF8	14		54	M		real-time PCR		IHC staining

MS	mitral stenosis
CAD	Coronary artery disease
Nor	Normal heart
HCM	Hypertrophic cardiomyopathy
HF	Heart failure

Table S2. Number of raw read-count and clean read-count confirming the quality of RNA-seq

sample	library	raw_reads	raw_bases	clean_reads	clean_bases	error_rate	Q20	Q30	GC_pct
WT_5	FRAS230068616-1r	40281110	6.04G	38700750	5.81G	0.03	97.77	93.6	44.57
WT_6	FRAS230068617-2r	46489676	6.97G	44824852	6.72G	0.02	98.03	94.32	45.1
WT_7	FRAS230068618-2r	45390148	6.81G	44287302	6.64G	0.03	97.91	94.06	44.75
WT_10	FRAS230068621-2r	42250282	6.34G	41024136	6.15G	0.03	97.93	93.99	44.95
circSLC_1	FRAS230068622-2r	43193682	6.48G	40946948	6.14G	0.03	97.85	93.82	44.91
circSLC_3	FRAS230068623-2r	40181054	6.03G	38257974	5.74G	0.03	97.96	94.06	45.11
circSLC_6	FRAS230068626-2r	44677264	6.7G	42745430	6.41G	0.03	97.82	93.75	44.22
circSLC_10	FRAS230068630-1r	44180900	6.63G	42424948	6.36G	0.02	98.11	94.39	44.6

Table S3. Distribution of ratio of total-read to reference genes, analyzing the deviation of different samples.

sample	total_reads	total_map	unique_map	multi_map	read1_map	read2_map	positive_map	negative_map	splice_map	unsplice_map	proper_map
WT_5	38700750	37685169 (97.38%)	27915371 (72.13%)	9769798 (25.24%)	13991382 (36.15%)	13923989 (35.98%)	13940704 (36.02%)	13974667 (36.11%)	8842141 (22.85%)	19073230 (49.28%)	27146366 (70.14%)
WT_6	44824852	43718424 (97.53%)	32941583 (73.49%)	10776841 (24.04%)	16489015 (36.79%)	16452568 (36.7%)	16454939 (36.71%)	16486644 (36.78%)	10683412 (23.83%)	22258171 (49.66%)	32143968 (71.71%)
WT_7	44287302	43065684 (97.24%)	31636560 (71.43%)	11429124 (25.81%)	15848527 (35.79%)	15788033 (35.65%)	15811050 (35.7%)	15825510 (35.73%)	9981772 (22.54%)	21654788 (48.9%)	30926132 (69.83%)
WT_10	41024136	39908986 (97.28%)	29541644 (72.01%)	10367342 (25.27%)	14800399 (36.08%)	14741245 (35.93%)	14760156 (35.98%)	14781488 (36.03%)	9159906 (22.33%)	20381738 (49.68%)	28897656 (70.44%)
circSLC_1	40946948	39855353 (97.33%)	29749320 (72.65%)	10106033 (24.68%)	14910918 (36.42%)	14838402 (36.24%)	14867569 (36.31%)	14881751 (36.34%)	9154834 (22.36%)	20594486 (50.3%)	29034880 (70.91%)
circSLC_3	38257974	37367116 (97.67%)	28079475 (73.4%)	9287641 (24.28%)	14060400 (36.75%)	14019075 (36.64%)	14031223 (36.68%)	14048252 (36.72%)	9044432 (23.64%)	19035043 (49.75%)	27427554 (71.69%)
circSLC_6	42745430	41727436 (97.62%)	31105489 (72.77%)	10621947 (24.85%)	15589777 (36.47%)	15515712 (36.3%)	15550009 (36.38%)	15555480 (36.39%)	9084295 (21.25%)	22021194 (51.52%)	30369552 (71.05%)
circSLC_10	42424948	41531801 (97.89%)	30774822 (72.54%)	10756979 (25.36%)	15404688 (36.31%)	15370134 (36.23%)	15382661 (36.26%)	15392161 (36.28%)	9305332 (21.93%)	21469490 (50.61%)	30124006 (71.01%)

Table S4. Pearson correlation between samples

	WT_5	WT_6	WT_7	WT_10	circSLC_1	circSLC_3	circSLC_6	circSLC_10
circSLC_10	0.941	0.942	0.963	0.948	0.952	0.922	0.908	1
circSLC_6	0.935	0.927	0.901	0.93	0.919	0.928	1	0.908
circSLC_3	0.947	0.935	0.92	0.945	0.935	1	0.928	0.922
circSLC_1	0.949	0.961	0.942	0.961	1	0.935	0.919	0.952
WT_10	0.957	0.963	0.943	1	0.961	0.945	0.93	0.948
WT_7	0.94	0.943	1	0.943	0.942	0.92	0.901	0.963
WT_6	0.947	1	0.943	0.963	0.961	0.935	0.927	0.942
WT_5	1	0.947	0.94	0.957	0.949	0.947	0.935	0.941

R^2: 1.000, 0.975, 0.950, 0.925

Table S5. MS analysis revealing proteins precipitated by anti-Slc8a1 antibody

Identified Proteins	Access No	ID	MW	vector	anti-Slc-604
Acylglycerol kinase, mitochondrial	E9PC15 (+1)	AGK	44 kDa	0	8
Apoptosis-inducing factor 1, mitochondrial	O95831	AIFM1	67 kDa	10	21
A-kinase anchor protein 1, mitochondrial	Q92667	AKAP1	97 kDa	0	5
Aldehyde dehydrogenase X, mitochondrial	P30837	ALDH1B1	57 kDa	0	11
ATP synthase subunit alpha, mitochondrial	P25705	ATP5F1A	60 kDa	17	56
ATP synthase subunit beta, mitochondrial	P06576	ATP5F1B	57 kDa	6	58
ATP synthase subunit gamma, mitochondrial	P36542	ATP5F1C	33 kDa	10	22
ATP synthase F(0) complex subunit B1, mitochondrial	P24539	ATP5PB	29 kDa	0	15
ATP synthase subunit d, mitochondrial	O75947	ATP5PD	18 kDa	0	11
ATP synthase subunit O, mitochondrial	P48047	ATP5PO	23 kDa	0	18
Cytochrome c1, heme protein, mitochondrial	P08574	CYC1	35 kDa	0	8
28S ribosomal protein S29, mitochondrial	P51398	DAP3	46 kDa	0	7
DnaJ homolog subfamily A member 3, mitochondrial	Q96EY1	DNAJA3	52 kDa	0	5
Stress-70 protein, mitochondrial	P38646	HSPA9	74 kDa	10	22
60 kDa heat shock protein, mitochondrial	P10809	HSPD1	61 kDa	19	50
Leucine-rich PPR motif-containing protein, mitochondrial	P42704	LRPPRC	158 kDa	6	31
Calcium uniporter protein, mitochondrial	Q8NE86	MCU	40 kDa	0	7
39S ribosomal protein L11, mitochondrial	Q9Y3B7	MRPL11	21 kDa	0	9
39S ribosomal protein L15, mitochondrial	Q9P015	MRPL15	33 kDa	0	5
28S ribosomal protein S22, mitochondrial	G5E9V5 (+2)	MRPS22	41 kDa	0	7
28S ribosomal protein S7, mitochondrial	J3QLS3 (+1)	MRPS7	32 kDa	0	6
Mitochondrial carrier homolog 2	Q9Y6C9	MTCH2	33 kDa	0	9
NADH dehydrogenase 1 alpha subcomplex subunit 10, mitochondrial	A0A087WXC5 (+2)	NDUFA10	41 kDa	0	12
NADH dehydrogenase iron-sulfur protein 3, mitochondrial	O75489	NDUFS3	30 kDa	0	9
Pyruvate dehydrogenase E1 component subunit beta, mitochondrial	P11177	PDHB	39 kDa	5	16
Serine/threonine-protein phosphatase PGAM5, mitochondrial	Q96HS1	PGAM5	32 kDa	6	10
DNA-directed RNA polymerase, mitochondrial	O00411	POLRMT	139 kDa	0	10
Pentatricopeptide repeat domain-containing protein 3, mitochondrial	Q96EY7	PTCD3	79 kDa	0	8
Tricarboxylate transport protein, mitochondrial	P53007	SLC25A1	34 kDa	0	14
Mitochondrial dicarboxylate carrier	Q9UBX3	SLC25A10	31 kDa	0	11
Mitochondrial 2-oxoglutarate/malate carrier protein	I3L1P8 (+1)	SLC25A11	32 kDa	0	17
Calcium-binding mitochondrial carrier protein Aralar2	Q9UJS0	SLC25A13	74 kDa	7	23
Phosphate carrier protein, mitochondrial	F8VVM2	SLC25A3	36 kDa	0	26
Stomatin-like protein 2, mitochondrial	Q9UJZ1	STOML2	39 kDa	0	22
Threonine--tRNA ligase, mitochondrial	Q9BW92	TARS2	81 kDa	0	5
Mitochondrial import inner membrane translocase subunit TIM44	O43615	TIMM44	51 kDa	0	10
Mitochondrial import inner membrane translocase subunit TIM50	Q3ZCQ8	TIMM50	40 kDa	5	16
Mitochondrial import receptor subunit TOM40	O96008	TOMM40	38 kDa	0	10
Elongation factor Tu, mitochondrial	P49411	TUFM	50 kDa	21	48
Cytochrome b-c1 complex subunit 2, mitochondrial	P22695	UQCRC2	48 kDa	7	15
Outer mitochondrial membrane protein porin 2	A0A0A0MR02 (+1)	VDAC2	30 kDa	0	20

Table S6. MS analysis revealing proteins precipitated by His-column

Identified Proteins	Access No	ID	MW	vector	His-Slc-604
Alanine--tRNA ligase, mitochondrial	Q5JTZ9	AARS2	107 kDa	7	15
Alpha-aminoadipic semialdehyde synthase, mitochondrial	Q9UDR5	AASS	102 kDa	0	37
ATP-binding cassette sub-family B member 7, mitochondrial	A0A5F9ZA98	ABCB7	83 kDa	0	11
Mycophenolic acid acyl-glucuronide esterase, mitochondrial	Q9NUJ1	ABHD10	34 kDa	0	11
Complex I assembly factor ACAD9, mitochondrial	Q9H845	ACAD9	69 kDa	0	15
Aconitate hydratase, mitochondrial	A2A274	ACO2	88 kDa	0	44
Acyl-coenzyme A thioesterase 2, mitochondrial	A0A087X0W7	ACOT2	46 kDa	0	25
Acyl-coenzyme A thioesterase 9, mitochondrial	Q9Y305	ACOT9	50 kDa	0	24
Malonate--CoA ligase ACSF3, mitochondrial	Q4G176	ACSF3	64 kDa	0	20
Adenylate kinase 4, mitochondrial	P27144	AK4	25 kDa	0	8
A-kinase anchor protein 1, mitochondrial	Q92667	AKAP1	97 kDa	0	12
5-aminolevulinate synthase, nonspecific, mitochondrial	P13196	ALAS1	71 kDa	0	10
Branched-chain-amino-acid aminotransferase, mitochondrial	O15382	BCAT2	44 kDa	0	5
Mitochondrial potassium channel	Q96ER9	CCDC51	46 kDa	0	5
Clustered mitochondria protein homolog	A0A494C0R8	CLUH	150 kDa	0	39
Atypical kinase COQ8A, mitochondrial	Q8NI60	COQ8A	72 kDa	0	5
Cytochrome c oxidase subunit 4 isoform 1, mitochondrial	P13073	COX4I1	20 kDa	0	11
Oxygen-dependent coproporphyrinogen-III oxidase, mitochondrial	P36551	CPOX	50 kDa	0	28
Carnitine O-palmitoyltransferase 2, mitochondrial	A0A1B0GTB8	CPT2	71 kDa	0	8
Cytochrome c1, heme protein, mitochondrial	P08574	CYC1	35 kDa	0	21
28S ribosomal protein S29, mitochondrial	P51398	DAP3	46 kDa	7	32
Aspartate--tRNA ligase, mitochondrial	Q6PI48	DARS2	74 kDa	0	61
Probable 2-oxoglutarate dehydrogenase E1 component DHKTD1, mitochondrial	Q96HY7	DHTKD1	103 kDa	0	30
Dihydrolipoyl dehydrogenase, mitochondrial	P09622	DLD	54 kDa	0	14
Probable glutamate--tRNA ligase, mitochondrial	Q5JPH6	EARS2	59 kDa	5	12
Electron transfer flavoprotein-ubiquinone oxidoreductase, mitochondrial	Q16134	ETFDH	68 kDa	0	22
Phenylalanine--tRNA ligase, mitochondrial	O95363	FARS2	52 kDa	0	9
FAST kinase domain-containing protein 2, mitochondrial	Q9NYY8	FASTKD2	81 kDa	0	14
FAST kinase domain-containing protein 5, mitochondrial	Q7L8L6	FASTKD5	87 kDa	0	21
Glycine dehydrogenase (decarboxylating), mitochondrial	P23378	GLDC	113 kDa	0	29
Glutaminase kidney isoform, mitochondrial	O94925	GLS	73 kDa	7	25
Glutamate dehydrogenase 1, mitochondrial	P00367	GLUD1	61 kDa	0	33
Aspartate aminotransferase, mitochondrial	P00505	GOT2	48 kDa	0	15
tRNA modification GTPase GTPBP3, mitochondrial	Q969Y2	GTPBP3	52 kDa	0	7
Hydroxyacylglutathione hydrolase, mitochondrial	Q16775	HAGH	34 kDa	0	13
Histidine triad nucleotide-binding protein 2, mitochondrial	Q9BX68	HINT2	17 kDa	0	10
Isoleucine--tRNA ligase, mitochondrial	Q9NSE4	IARS2	114 kDa	0	79
Isocitrate dehydrogenase [NADP], mitochondrial	P48735	IDH2	51 kDa	6	24
Isocitrate dehydrogenase [NAD] subunit, mitochondrial	A0A087WZN1 (+1)	IDH3B	42 kDa	0	11
Isocitrate dehydrogenase [NAD] subunit gamma, mitochondrial	P51553	IDH3G	43 kDa	0	11
Probable leucine--tRNA ligase, mitochondrial	Q15031	LARS2	102 kDa	0	55
Mitochondrial proton/calcium exchanger protein	O95202	LETM1	83 kDa	7	31
Lon protease homolog, mitochondrial	K7EJE8 (+1)	LONP1	93 kDa	0	35
Methionine--tRNA ligase, mitochondrial	Q96GW9	MARS2	67 kDa	0	30
Malonyl-CoA-acyl carrier protein transacylase, mitochondrial	Q8IVS2	MCAT	43 kDa	0	44
Methylcrotonoyl-CoA carboxylase beta chain, mitochondrial	Q9HCC0	MCCC2	61 kDa	0	25
Mitochondrial intermediate peptidase	Q99797	MIPEP	81 kDa	0	29
Methylmalonyl-CoA mutase, mitochondrial	P22033	MMUT	83 kDa	0	8
39S ribosomal protein L10, mitochondrial	Q7Z7H8	MRPL10	29 kDa	0	7
39S ribosomal protein L13, mitochondrial	Q9BYD1	MRPL13	21 kDa	0	8
39S ribosomal protein L3, mitochondrial	E7ETU7 (+2)	MRPL3	42 kDa	0	9
39S ribosomal protein L37, mitochondrial	Q9BZE1	MRPL37	48 kDa	7	33
39S ribosomal protein L38, mitochondrial	Q96DV4	MRPL38	45 kDa	0	7
39S ribosomal protein L4, mitochondrial	K7ES61 (+1)	MRPL4	34 kDa	0	9
28S ribosomal protein S18b, mitochondrial	Q9Y676	MRPS18B	29 kDa	0	10
28S ribosomal protein S2, mitochondrial	Q9Y399	MRPS2	33 kDa	0	12
28S ribosomal protein S27, mitochondrial	Q92552	MRPS27	48 kDa	5	22
28S ribosomal protein S5, mitochondrial	P82675	MRPS5	48 kDa	0	18
28S ribosomal protein S7, mitochondrial	J3QLS3 (+1)	MRPS7	32 kDa	6	21
Poly(A) RNA polymerase, mitochondrial	Q9NVV4	MTPAP	66 kDa	0	9
NAD kinase 2, mitochondrial	Q4G0N4	NADK2	49 kDa	0	8
Probable asparagine--tRNA ligase, mitochondrial	Q96I59	NARS2	54 kDa	0	12
NADH dehydrogenase 1 alpha subcomplex subunit 9, mitochondrial	Q16795	NDUFA9	43 kDa	0	23
NADH dehydrogenase iron-sulfur protein 2, mitochondrial	O75306	NDUFS2	53 kDa	0	38
NADH dehydrogenase iron-sulfur protein 6, mitochondrial	O75380	NDUFS6	14 kDa	0	8
2-oxoglutarate dehydrogenase, mitochondrial	Q02218	OGDH	116 kDa	0	47
Pyruvate carboxylase, mitochondrial	P11498	PC	130 kDa	0	19
Phosphoenolpyruvate carboxykinase [GTP], mitochondrial	Q16822	PCK2	71 kDa	0	8
Pyruvate dehydrogenase E1 component subunit alpha, somatic form, mitochondrial	P08559	PDHA1	43 kDa	0	8
[Pyruvate dehydrogenase [acetyl-transferring]]-phosphatase 1, mitochondrial	Q9P0J1	PDP1	61 kDa	0	10
Pyruvate dehydrogenase phosphatase regulatory subunit, mitochondrial	Q8NCN5	PDPR	99 kDa	0	9
Phosphatidylserine decarboxylase proenzyme, mitochondrial	Q9UG56	PISD	47 kDa	0	12
Presequence protease, mitochondrial	Q5JRX3	PITRM1	117 kDa	0	64
Mitochondrial-processing peptidase subunit alpha	Q10713	PMPCA	58 kDa	0	28
DNA-directed RNA polymerase, mitochondrial	O00411	POLRMT	139 kDa	11	53
Thioredoxin-dependent peroxide reductase, mitochondrial	P30048	PRDX3	28 kDa	0	34
Mitochondrial Rho GTPase 2	Q8IXI1	RHOT2	68 kDa	0	9
Mitochondrial mRNA pseudouridine synthase RPUSD3	Q6P087	RPUSD3	38 kDa	0	7
Succinate dehydrogenase flavoprotein subunit, mitochondrial	P31040	SDHA	73 kDa	0	72
Serine hydroxymethyltransferase, mitochondrial	P34897	SHMT2	56 kDa	0	41
Calcium-binding mitochondrial carrier protein Aralar1	O75746	SLC25A12	75 kDa	0	9
Succinate--CoA ligase subunit alpha, mitochondrial	P53597	SUCLG1	36 kDa	0	8
ATP-dependent RNA helicase SUPV3L1, mitochondrial	Q8IYB8	SUPV3L1	88 kDa	0	22
Threonine--tRNA ligase, mitochondrial	Q9BW92	TARS2	81 kDa	5	27
Dimethyladenosine transferase 2, mitochondrial	Q9H5Q4	TFB2M	45 kDa	0	9
Mitochondrial import inner membrane translocase subunit TIM50	Q3ZCQ8	TIMM50	40 kDa	11	16
Trimethyllysine dioxygenase, mitochondrial	Q9NVH6	TMLHE	50 kDa	0	12
Mitochondrial import receptor subunit TOM22 homolog	Q9NS69	TOMM22	16 kDa	0	11
Heat shock protein 75 kDa, mitochondrial	Q12931	TRAP1	80 kDa	12	45
tRNA (adenine(58)-N(1))-methyltransferase, mitochondrial	Q9BVS5	TRMT61B	53 kDa	0	6
Cytochrome b-c1 complex subunit 1, mitochondrial	P31930	UQCRC1	53 kDa	0	13
Cytochrome b-c1 complex subunit 2, mitochondrial	P22695	UQCRC2	48 kDa	6	34
Tryptophan--tRNA ligase, mitochondrial	Q9UGM6	WARS2	40 kDa	0	17
Tyrosine--tRNA ligase, mitochondrial	Q9Y2Z4	YARS2	53 kDa	7	33
